# The ubiquitin-conjugating enzyme CDC34 is essential for cytokinesis in contrast to putative subunits of a SCF complex in *Trypanosoma brucei*

**DOI:** 10.1371/journal.pntd.0005626

**Published:** 2017-06-13

**Authors:** Federico Rojas, Joanna Koszela, Jacqueline Búa, Briardo Llorente, Richard Burchmore, Manfred Auer, Jeremy C. Mottram, María Teresa Téllez-Iñón

**Affiliations:** 1 Instituto de Investigaciones en Ingeniería Genética y Biología Molecular (INGEBI-CONICET), Buenos Aires, Argentina; 2 Institute of Quantitative Biology Biochemistry and Biotechnology, School of Biological Sciences, University of Edinburgh, King’s Buildings, Edinburgh, United Kingdom; 3 Instituto Nacional de Parasitología ‘Dr. M. Fatala Chabén’, A.N.L.I.S., ‘Dr. Carlos G. Malbrán’, Buenos Aires, Argentina; 4 Institute of Infection, Immunity and Inflammation, College of Medical, Veterinary and Life Sciences, University of Glasgow, Glasgow, United Kingdom; 5 Centre for Immunology and Infection, Department of Biology, University of York, York, United Kingdom; Liverpool School of Tropical Medicine, UNITED KINGDOM

## Abstract

The ubiquitin-proteasome system is a post-translational regulatory pathway for controlling protein stability and activity that underlies many fundamental cellular processes, including cell cycle progression. Target proteins are tagged with ubiquitin molecules through the action of an enzymatic cascade composed of E1 ubiquitin activating enzymes, E2 ubiquitin conjugating enzymes, and E3 ubiquitin ligases. One of the E3 ligases known to be responsible for the ubiquitination of cell cycle regulators in eukaryotes is the SKP1-CUL1-F-box complex (SCFC). In this work, we identified and studied the function of homologue proteins of the SCFC in the life cycle of *Trypanosoma brucei*, the causal agent of the African sleeping sickness. Depletion of trypanosomal SCFC components TbRBX1, TbSKP1, and TbCDC34 by RNAi resulted in decreased growth rate and contrasting cell cycle abnormalities for both procyclic (PCF) and bloodstream (BSF) forms. Depletion of TbRBX1 in PCF cells interfered with kinetoplast replication, whilst depletion of TbSKP1 arrested PCF and BSF cells in the G1/S transition. Silencing of TbCDC34 in BSF cells resulted in a block in cytokinesis and caused rapid clearance of parasites from infected mice. We also show that TbCDC34 is able to conjugate ubiquitin *in vitro* and *in vivo*, and that its activity is necessary for *T*. *brucei* infection progression in mice. This study reveals that different components of a putative SCFC have contrasting phenotypes once depleted from the cells, and that TbCDC34 is essential for trypanosome replication, making it a potential target for therapeutic intervention.

## Author summary

African sleeping sickness is a neglected tropical disease caused by infection with the protozoan parasite *Trypanosoma brucei*, which is transmitted to humans by tsetse flies (*Glossina* genus). Treatment of the disease is complex and relies on limited pharmaceutical options. Understanding how *T*. *brucei* regulates cell cycle progression at a molecular level when alternating between the mammalian host and the insect vector could lead to better therapies. In this study, we examined different *T*. *brucei* proteins with homology to components of the SKP1-CUL1-F-box ubiquitin ligase complex (SCFC), previously characterized in other eukaryotes as a regulator of cell cycle progression. We found that depletion of the homologues of a putative SCFC cause *T*. *brucei* to develop abnormally, generating different phenotypes of the mammalian and insect stages. Interestingly, depletion of the ubiquitin conjugating enzyme TbCDC34 arrest cells in a pre-cytokinesis stage, indicating that this protein is essential for cytokinesis. In addition to improving our fundamental understanding of the molecular regulation underlying the sophisticated life cycle of *T*. *brucei*, this work pinpoints a potential target for drug development against trypanosomiasis.

## Introduction

Transition from one cell cycle stage to the next is achieved in eukaryotes through mechanisms that provide an all-or-none cyclin-dependent kinases (CDKs) activation. The activity of these enzymes is regulated by several mechanisms, including association with regulatory subunits (cyclins), phosphorylation and dephosphorylation and interaction with CDK inhibitors (CKIs) [1, 2]. Levels of cyclins (CYC), CKIs and many other cell cycle regulators oscillate during the cell cycle as a result of periodic proteolysis, generating a unidirectional control. These proteins are targeted for degradation by polyubiquitination, i.e. the attachment of multiple copies of ubiquitin. This process is initiated by an ubiquitin-activating enzyme (E1), which then transfers ubiquitin to an active cysteine residue of an ubiquitin-conjugating enzyme (E2) as a thioester linkage. Although E2s can attach ubiquitin directly to a lysine residue in a substrate, most physiological ubiquitination reactions require an ubiquitin ligase, or E3 [3]. Once the substrate is polyubiquitinated by an E3, it is then recognized and degraded by the 26S proteasome. The specificity of ubiquitin-dependent proteolysis derives from the many hundreds of E3 ubiquitin ligases that recognize particular substrates through interaction domains [4].

Two E3 enzymes that comprise multiprotein components are of particular importance for cell cycle progression: the SKP1-CULLIN1-F-box complex (SCFC) and the anaphase promoting complex or cyclosome (APC/C) [5]. The SCFC consists of three invariant components including SKP1, CULLIN1, RBX1/ROC1/HRT1, the E2 ubiquitin conjugating enzyme CDC34 and various F-box proteins [6]. SKP1 and CULLIN1 are required for the structural organization of the SCFC, whereas RBX1 is a RING (Really Interesting New Gene)-finger protein that interacts with E2-ubiquitin-conjugating enzymes. F-box proteins play a unique role in SCFC function, as they interact with SKP1 via their F-box motif and simultaneously bind specific substrates to the SCFC [7]. Each F-box is responsible for recruiting individual hyperphosphorylated substrates to various SCFCs that differ in their F-box adapter protein. Once the substrate is recruited, CDC34 transfers ubiquitin molecules onto lysine residues in the substrate, building polyubiquitin chains. Polyubiquitinated proteins are recognised and degraded by the 26*S* proteasome.

The protozoan parasite *Trypanosoma brucei* is the etiological agent of African trypanosomiasis in humans (sleeping sickness) and cattle (nagana). This parasite has a complex life cycle that alternates between the mammalian hosts and the insect vectors of the *Glossina* genus (Tsetse flies). The dividing procyclic form (PCF) in insects and the long slender bloodstream form (BSF) in mammals follow sequential G1, S, G2, and M phases [8]. A single mitochondrion in each cell contains a DNA complex termed the kinetoplast, which divides co-ordinately with the nucleus [9, 10].

Previous work on the 26S proteasome of *T*. *brucei* suggested a role for the ubiquitin-mediated degradation of trypanosomatid proteins in the control of cell cycle [11, 12] and the major effect that can be achieved by targeting this enzyme [13]. In order to investigate the role of the ubiquitination machinery in the cell cycle control of *T*. *brucei*, we studied the function of trypanosomal homologous genes of the SCFC. Our results reveal that the identified proteins are necessary for the normal proliferation of *T*. *brucei* and that knockdown of these genes generate contrasting phenotypes. The apparent differential activities of these proteins may indicate they don´t form a classic SCFC. Besides establishing the importance of these proteins for the growth of *T*. *brucei in vitro*, we also demonstrate that the ubiquitin-conjugating enzyme TbCDC34 is able to conjugate ubiquitin, being indispensable for the maintenance of infection in a mammalian host.

## Materials and methods

### Identification of SCFC subunit homologues in *T*. *brucei*

Protein sequences of yeast (*Saccharomyces cerevisiae*) and human (*Homo sapiens*) SCFC subunits were obtained from the Entrez Protein database using the NCBI web site (http://www.ncbi.nlm.nih.gov/sites/entrez?db=protein). Each sequence was used as a query to screen the kinetoplastid genome database at GeneDB (http://www.genedb.org/) for potential SCFC candidates. The identified subunit homologues are listed in Table 1. Sequences were aligned using Multiple Sequence Alignment by CLUSTALW (http://align.genome.jp). Parameters used for these alignments were: gap open penalty = 10; gap extension penalty = 0.05.

**Table 1:** Homologous proteins of the yeast and human SCFC were identified in the genomes of *Trypanosoma brucei*, *Trypanosoma cruzi* and *Leishmania major* by reciprocal best blast hits.

Protein	PFAM Domains	*T*.*brucei*	GeneDB	E-value	*T*.*cruzi*	GeneDB	*L*. *major*	GeneDB
**SKP1**	**PF03931**	**TbSKP1**	**Tb927.11.6130**	**3,5E-24**	**TcSKP1**	**Tc00.1047053506297.350**	**LmSKP1**	**LmjF11.1210**
	**PF01466**					**Tc00.1047053508041.10**		
**CDC53/CULLIN1**	**PF00888**	**TbCULLIN1**	**Tb927.8.5970**	**2,3E-43**	**TcCULLIN1**	**Tc00.1047053511075.40**	**LmCULLIN1**	**LmjF24.2290**
**RBX1**	**PF00097**	**TbRBX1**	**Tb927.10.1810**	**2,8E-38**	**TcRBX1**	**Tc00.1047053503965.20**	**LmRBX1**	**LmjF.0023**
						**Tc00.1047053506495.9**		
**CDC34**	**PF00179**	**TbCDC34**	**Tb927.11.14200**	**7,4E-31**	**TcCDC34**	**Tc00.1047053511727.40**	**LmCDC34**	**LmjF32.0960**

### Cell lines

The procyclic form *T*. *brucei* strain 29–13 [14] was cultured in SDM-79 medium at 28°C supplemented with (v/v) tetracycline-deficient fetal bovine serum (BD Biosciences, Franklin Lakes, New Jersey, USA) and 3.5 mg/ml hemin. Bloodstream form cell line 90–13 [14] was grown at 37°C with 5% CO_2_ supplied in HMI-9 medium containing 10% fetal bovine serum. To maintain the T7 RNA polymerase and tetracycline repressor gene constructs within the cells, 15 μg/ml G418 and 50 μg/ml hygromycin B were added to the SDM79 medium for the 29–13 cell line, whereas 2.5 μg/ml G418 and 5 μg/ml hygromycin B were added to the HMI-9 medium for the 90–13 cell line.

### Plasmid constructs for protein overexpression and RNA interference

Primers for amplification of an RNA interference (RNAi) target fragment were designed using the RNAit software tool (http://trypanofan.path.cam.ac.uk/software/RNAit.html). RNAi experiments were designed to knockdown the SCFC subunit homologues identified in *T*. *brucei* (S1 Table). RNAi target fragments were amplified by PCR using Phusion DNA polymerase (Finnzymes, Espoo, Finland) and genomic DNA template from 29–13 PCF cells with the primers listed in S1 Table that amplify: nucleotides 1116–1613 for TbCULLIN1, nucleotides 16–419 for TbSKP1, nucleotides 3–307 for TbRBX1 and nucleotides 31–444 for TbCDC34. The corresponding DNA fragments were ligated into the pZJM vector [15] by replacing the α-tubulin fragment. The resulting RNAi constructs were linearized with NotI enzyme and introduced into *T*. *brucei* cells by electroporation. Transfection of the *T*. *brucei* procyclic form was performed according to the procedure described in [14]. Transfection of bloodstream form was performed with an Amaxa Nucleofactor electroporator (Amaxa, Basel, Switzerland) using program X-001 and human T cell solution, using 2.5×10^7^ cells and 10 μg of DNA. After phleomycin selection, single transfected cells were cloned on 24-well plates, cultivated under phleomycin, and induced by tetracycline to synthesize the double-stranded RNA. Growth studies were initiated by diluting logarithmically growing cells to a starting density of 1x10^5^ cells/ml (BSF) or 1x10^6^ cells/ml (PCF). Cell density was measured with a Neubauer haemocytometer.

To ectopically express HA-tagged proteins, full-length TbCDC34 was amplified by PCR using the primers described in S1 Table from *T*. *brucei* 29–13 strain genomic DNA. The fragments were cloned in p2477/p2619 expressing vector [16] and transfected into *T*. *brucei* 29–13 or 90–13 strains.

To generate double mutants C84S/S86D of TbCDC34, a degenerate oligonucleotide encoding C84S and S86D was used with the complete gene cloned in TOPO as template for PCR mutagenesis using the Stratagene Quikchange mutagenesis (La Jolla, California, USA) kit as instructed by the manufacturer. All constructs were verified by standard sequencing methods (Macrogene, Seoul, Korea) prior to introduction into trypanosomes, and expression was further verified by western blotting where appropriate.

To generate the endogenous modified version of TbCDC34, part of the open reading frame of TbCDC34 and the 6HA from p2477-TbCDC34 was amplified, and together with the 3´untranslated region product were inserted into pENT6BTyYFP [16] between the HindIII and XbaI restriction sites, generating a modified version of the plasmid with a 6HA tag.

### Protein expression

6His-TbCDC34 and yeast CDC34∆C-His6 lacking the C-terminal 25 amino acids (provided by R. Deshaies) [17] were expressed in *Escherichia coli BL21*(DE3)+pLysE cells and purified by Ni-NTA (Qiagen, Venlo, Netherlands), according to the manufacturer.

### Ubiquitin pull-down assay and *in vitro* gel-based ubiquitination assay

Ubiquitinated proteins were isolated using UbiQapture-Q kit (Enzo Life Sciences, Farmingdale, New York, USA) according to the manufacturer´s instruction. Ubiquitinated proteins were isolated from the total cell lysates (25 μg total protein of parasites overexpressing TbCDC34 wild-type or mutated version) with 40 μl of UbiQapture-Q matrix by rotating samples for 4 hours at 4°C. After washing four times, captured proteins were eluted with 2X SDS-PAGE loading buffer and analyzed by western blotting using anti-HA antibody (Sigma-Aldrich, St. Louis, Missouri, USA).

To analyze TbCDC34 autoubiquitination and the formation of the thioester bond between TbCDC34 and human ubiquitin, the Ubiquitin activating kit (Enzo Life Sciences, Farmingdale, New York, USA) was used. 4 μM of purified wild-type or Cys mutant 6His-TbCDC34 or 6His-HsCdc34 were mixed with 100 nM of Uba1 and 2.5 μM of human ubiquitin in ubiquitination buffer, (50 μl total volume), with or without 5 mM Mg-ATP. Reactions were incubated at 37°C for 3 hours with gentle mixing, and were stopped by addition of an equal volume of 2× SDS-PAGE sample buffer and boiled for 5 min. Samples were analyzed by SDS-PAGE and western blotting using an anti-ubiquitin antibody (Enzo Life Sciences, Farmingdale, New York, USA) or an anti-6His antibody (Roche, Indianapolis, IN, USA) followed by detection with an infrared-coupled anti-mouse secondary antibody (Li-Cor Biosciences, Cambridge, UK). The cell permeable Cdc34 Inhibitor, CC0651, was purchased from Calbiochem.

### Protein electrophoresis and western blotting

Trypanosomes were harvested and washed twice in PBS. Pellets (1x10^7^ cells) were lysed in 100 μl of boiling SDS sample buffer (10% [v/v] glycerol, 3% [w/v] SDS, 0.01% [w/v] bromophenol blue and 50 mM Tris–HCl [pH: 6.8] with or without 100 mM dithiothreitol [DTT]) and resolved by SDS–PAGE on 10%, 12.5% or gradient 4–12% SDS–polyacrylamide mini gels. The proteins were electrophoretically transferred onto polyvinylidene fluoride (PVDF) membranes using a wet transfer tank (BioRad, Hercules, California, USA). For analysis of the *in vitro* ubiquitination assay, proteins were transferred to a 0.2 μm nitrocellulose membrane with a Trans Blot SD semi-dry transfer system (BioRad, Hercules, California, USA). Non-specific binding was blocked with Tris-buffered saline with Tween-20 (TBST) (137 mM NaCl, 2.7 mM KCl, 25 mM Tris base [pH: 7.4] and 0.2% Tween-20) supplemented with 5% milk. Commercial polyclonal anti-HA antibody was used at 1:1000 (Sigma-Aldrich, St. Louis, Missouri, USA), anti-ubiquitin antibody at 1:500 (Enzo Life Sciences, Farmingdale, New York, USA) and anti-6His antibody (Roche, Indianapolis, IN, USA) at 1:500. Incubations with secondary anti-IgG rabbit or anti-IgG mouse horseradish peroxidase conjugates (Sigma-Aldrich, St. Louis, Missouri, USA) were performed at 8,000-fold dilution in TBST with 1% BSA, while incubations with IRDye 800CW Donkey anti-mouse secondary antibody (Li-Cor Biosciences, Cambridge, UK) were performed at 1:20,000 dilution in TBST with 1% BSA. Detection was performed by chemiluminescence with ECL (GE Healthcare, Little Chalfont, Buckinghamshire, UK) on BioMaxMR film (Kodak, Rochester, New York, USA) or by fluorescence scanning using Odyssey CLx Infrared Imaging System and analyzed using Image Studio software (Li-Cor Biosciences, Cambridge, UK).

### Transcripts analysis

1x10^8^ cells were harvested at 3450 x g for 10 min at 4°C and washed with ice-cold PBS. Cells were frozen in dry ice for 1 min and total RNA was extracted using Trizol reagent (Invitrogen, Carlsbad, California, USA) according to the manufacturer’s instructions. Purified RNAs were quantified by spectroscopy using a Nanodrop ND-1000 spectrophotometer (Thermo Scientific, Massachusetts, USA) and RNA integrity was evaluated by agarose gel electrophoresis. RNA samples were treated with RNase-free DNase I (Promega, Fitchburg, Wisconsin, USA) and the Superscript III reverse transcriptase kit (Invitrogen, Carlsbad, California, USA) was used to generate cDNA according to the manufacturer’s instructions, using 50 pmol of oligo dTs and 5 μg of total RNA. The mRNA abundance of the selected genes was evaluated by quantitative reverse transcription PCR (RT-qPCR) using the primers listed in S1 Table. The GPI-anchor transamidase subunit 8 (GPI8) gene (Tb10.61.3060) of *T*. *brucei* was used as an endogenous control for gene normalization. qPCR reactions were performed in a total volume of 20 μl using Power SYBR Green PCR Master Mix (Applied Biosystems, Foster City, California, USA) on a Rotor-Gene 6000 instrument (Corbett Life Science, Australia) with 500 nM of each specific sense and anti-sense primers. Cycling conditions were 95°C for 10 min followed by 40 cycles of 30 s at 94°C, 30 s at 60°C, 30 s at 72°C. All gene fragments were amplified in triplicate from each biological replicate and the mean values were considered for further calculations using the standard curve method. All data were normalized to the level of the endogenous reference gene GPI8 and to WT expression values.

Northern blotting was carried out using standard procedures. RNA was extracted from 108 cells, and 1 μg RNA separated by gel electrophoresis prior to transfer to N-Hybond membrane. Specific probes were labelled with [α-^32^P] dCTP (3000 Ci/mmol) (10^9^ cpm/pmol, NEN) and signals were detected by scanning them with a PhosphorImager Storm 820 (Amersham Pharmacia Biotech, Sweden Biosciences).

### Cell cycle analysis

Cells were analyzed by flow cytometry for DNA content following induction of RNAi. Cells were collected by centrifugation at 600 x g (PCF) or 1500 x g (BSF) for 10 min and washed in cold PBS + 2 mM EDTA (ethylenediaminetetraacetic acid). The cell pellets were resuspended in 200 μl PBS + 2 mM EDTA and mixed with 1.5 ml of 70% ethanol in PBS and left overnight at 4°C. Cells were washed in PBS and incubated for 30 min at room temperature in 1 ml of PBS containing 10 mg/ml RNase A and 20 mg/ml propidium iodide. Fluorescence analysis was performed with the FACSCalibur flow cytometer (BD Biosciences, Franklin Lakes, New Jersey, USA). Flow cytometry data was fitted to G1, S, and G2/M curves using Dean-Jett-Fox model of FlowJo software (Tree Star, Ashland, Oregon, USA). To quantify nucleus-kinetoplast configurations, methanol-fixed smears were re-hydrated in PBS for 10 min and stained with DAPI (100 ng/ml) for 5 min. 250 cells were analyzed per slide.

### Infections in mice

Groups of five C3H/He and Balb/c one-month-old mice were inoculated with 1x10^6^ *T*. *brucei* 90–13 cells by intraperitoneal injection. Balb/c mice did not raise a significant parasitemia, therefore C3H/He were selected for further studies. Groups of five C3H/He mice were inoculated with 1x10^6^ BSF TbCDC34-RNAi parasites and different doxycycline (Sigma-Aldrich, St. Louis, Missouri, USA) drug treatments were performed. One group was administered doxycycline 1 mg/ml in 2.5% glucose at the beginning of the infection, orally and *ad libitum*. A control group only received 2.5% glucose. The course of the infection was evaluated by studying parasitemia and survival. Another experiment consisted in providing a group of five mice with 1 mg/ml doxycycline in water to induce TbCDC34 RNAi after 48 hours post-infection, when mice exhibited noticeable parasitemia (∼1.5x10^7^ cells/ml). A control group of five mice received water without doxycycline. Mice survival and parasitemia in peripheral blood obtained from tail bleeds were monitored each day. Experiments were repeated five times.

### Ethics statement

Animal trials in this manuscript were reviewed and approved by the Animal Care Committee of the Instituto Nacional de Parasitología “Dr. Mario Fatala Chaben”, Administración Nacionalde Laboratorios e Institutos de Salud “Dr. Carlos G. Malbrán” (Buenos Aires, Argentina), who evaluated the rationale, a clear scientific purpose and sample size of animals proposed. Animal studies were conducted in accordance with the Guide for the Care and Use of Laboratory Animals, 8th Edition (2011) and the NIH guidelines under an Institutional Animal Care and Use Committee-approved protocol from the Oregon Health and Sciences University.

### Statistical analysis

Statistical significance in parasite’s growth curves was determined by two-way repeated measures ANOVA test followed by Bonferony post-hoc analysis. Statistical significance in RT-qPCRs was determined by t-test analysis. Statistical analysis was performed using GraphPad Prism 5 (GraphPad Software, La Jolla, CA, USA).

## Results

### Identification of SCFC homologues in *T*. *brucei*

The core components of the SCFC of humans and yeast, SKP1, CULLIN1/CDC53, RBX1/ROC1 and the E2 enzyme CDC34, were used to screen the GeneDB databases of *T*. *brucei*, *T*. *cruzi* and *L*. *major* for homologous proteins. The search resulted in the identification of *T*. *brucei* candidate TbSKP1, TbCULLIN1, TbRBX1 and TbCDC34 proteins, all of which possess characteristic conserved domains (Table 1).

TbRBX1 shares 63% amino acid identity with its human counterpart (e-value 2,8E-38). The RING motif (aa. 41 to 95) is completely conserved in the amino acid sequences deduced for trypanosomatids. This domain is defined by a series of eight Cys, three His, and an Asp that binds zinc ions [18]. The identity of the sequence corresponding to the RING-H2 domain of TbRBX1 is 72% with its human counterpart (S1 Fig). TbSKP1 shows 33% identity (e-value 3,5E-24) with its human homologue. The highest amino acid conservation (57.8%) is found in the C-terminal region of the protein (S2 Fig). TbCULLIN1 shares 23% identity (e-value 2,3E-43) with human CULLIN1 (S3 Fig). The N-terminal domain involved in the interaction with SKP1 is the less conserved region of the protein. The cullin-homology (CH) domain, including residues 352 to 583, recruits the E2 enzyme and the RBX1 protein. This domain contains Arg442, which is important for the interaction with CDC34 in yeast [19]. The C-terminal region is the most conserved part of the protein (40%) and comprises the Lys residue that is modified by NEDD8 in humans (K686 in *T*. *brucei*). Neddylation of CULLIN1 increases ubiquitin ligase activity *in vitro* by facilitating the recruitment of E2 enzymes [20]. TbCDC34 possesses 20% identity with its human homologue (e-value 7,4E-31). The active site, the ubiquitin-conjugating (UBC) domain, contains the catalytic Cys (C84), the Ser involved in the formation of the ubiquitin chain (S86) and an insertion of 11 amino acids that differentiates CDC34 from other E2 enzymes (residues 93–104) [21, 22]. It also possesses a C-terminal extension that is essential for mediating the interaction with the SCFC in humans [23] and yeast [24] (S4 Fig).

### RNAi knockdown of SCFC subunits

**Fig 1:**
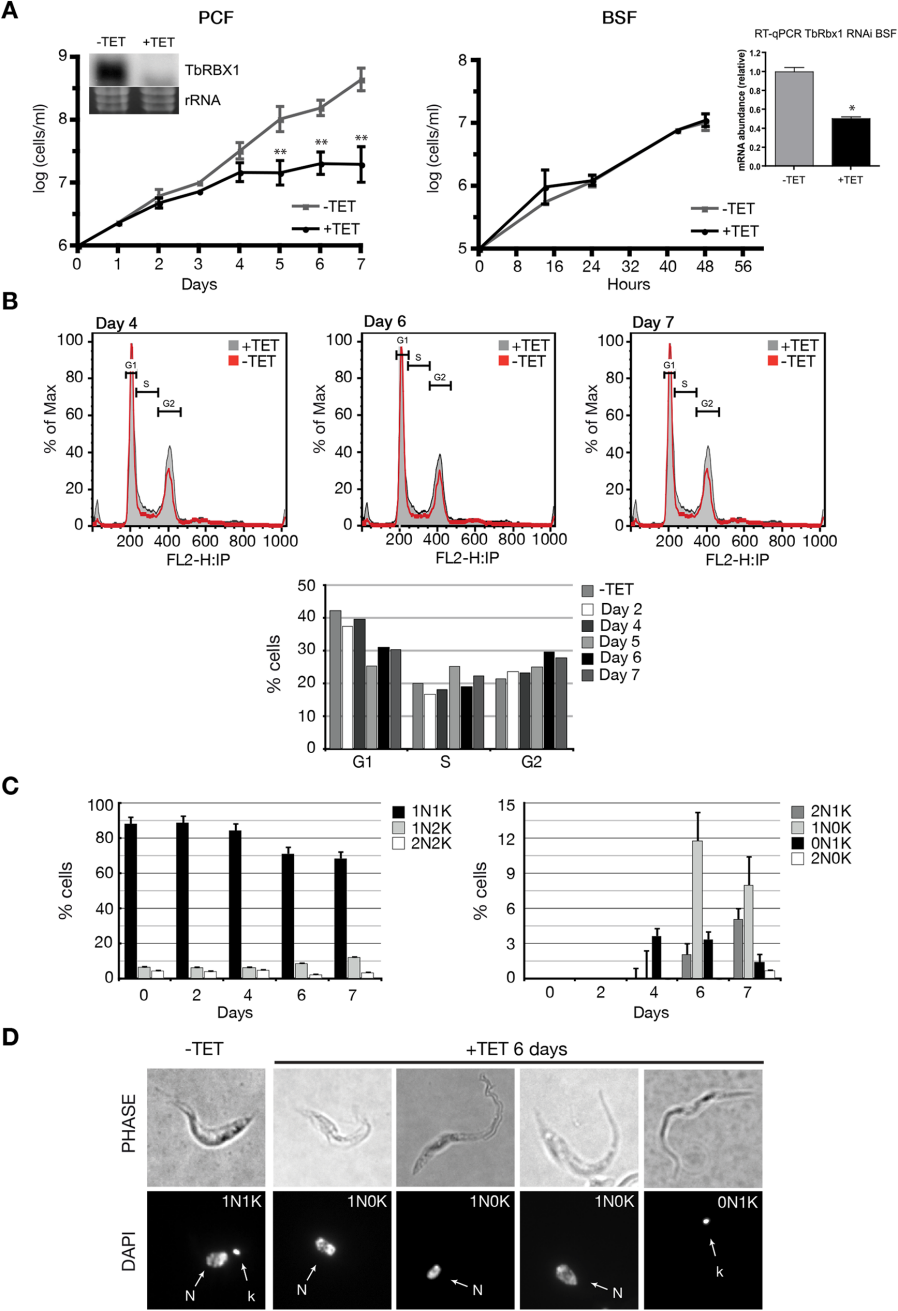
TbRBX1 depletion influences kDNA replication in the procyclic form of *T*.*brucei*. (A) Growth curves of PCF (left panel) and BSF (right panel) parasites after tetracycline-induced TbRBX1 RNAi. PCF and BSF parasites transfected with pZJM-TbRBX1-RNAi construct were cultured in the absence (-TET) or presence (+TET) of tetracycline (1μg/ml). Insets in the respective growth curves show northern blot (PCF) or RT-qPCR (BSF) experiments demonstrating the RNAi-mediated downregulation of TbRBX1 mRNA. Error bars represent the ± SEM of 3 biological replicates. *: *p≤ 0*.*01;* **: *p≤ 0*.*001*. (B) Flow cytometry analysis of TbRBX1-RNAi PCF parasites. The x-axis shows fluorescence intensity in the FL2-A channel. The bottom panel indicates percentage of cells in different cell cycle stages as determined using the FlowJo 7.5.5 software and the Dean-Jett-Fox model. (C) Nuclei (N) and kinetoplasts (K) configurations of TbRBX1-RNAi PCF parasites induced (+TET) with tetracycline for the indicated times. At least 200 cells were counted for each time point from 3 biological replicates. (D) Images of DAPI-stained cells viewed by phase-contrast and fluorescence microscopy. The effect of depleting TbRBX1 on cellular morphology, the nucleus (N) and kinetoplast (K) are shown.

**Fig 2:**
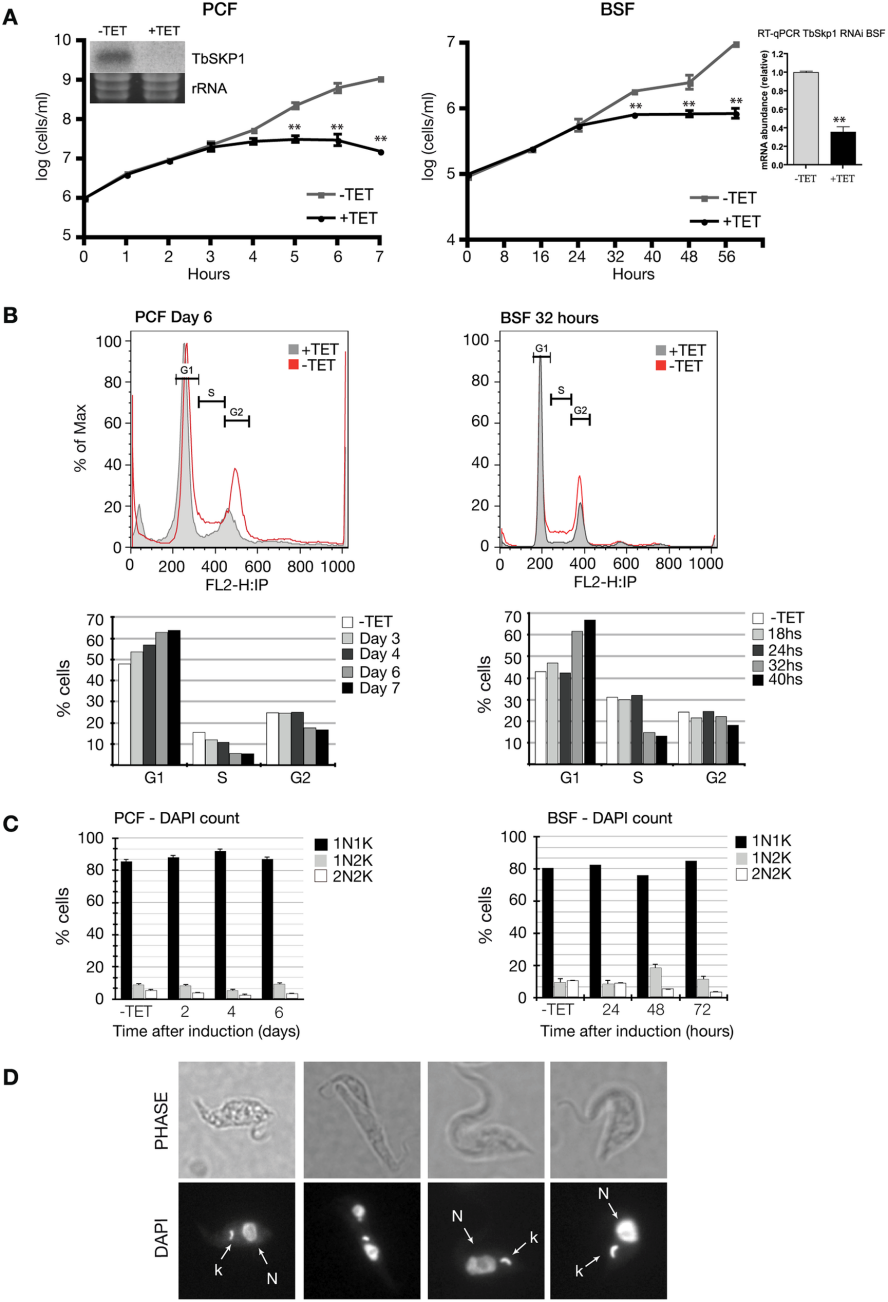
TbSKP1 promotes progression through the G1/S transition of the T. brucei cell cycle. (A) Growth curves of PCF TbSKP1-RNAi (left panel) and BSF TbSKP1-RNAi (right panel) parasites after tetracycline-induced RNAi. PCF or BSF parasites transfected with the pZJM-TbSKP1-RNAi construct were cultured in the absence (-TET) or presence (+TET) of tetracycline (1μg/ml). Insets in the respective growth curves show northern blot (PCF) or RT-qPCR (BSF) experiments demonstrating the RNAi-mediated downregulation of TbSKP1 mRNA. Error bars represent the ± SEM of 3 biological replicates. **: *p≤ 0*.*001*. (B) Flow cytometry analysis of PCF (left panels) and BSF (right panels) TbSKP1-RNAi parasites. The x-axis shows fluorescence intensity in the FL2-A channel. Bottom panel of each analysis shows the percentage of cells in each phase of the cell cycle as determined using Flowjo software. (C) DAPI analysis of nuclei and kinetoplast in PCF (left panel) and BSF (right panel) cells at different time points. These results are representative of at least three experiments. At least 100 cells were counted for each time point. (D) Images of BSF DAPI-stained cells viewed by phase-contrast and fluorescence microscopy. *N*: nucleus, *K*: kinetoplast.

**Fig 3:**
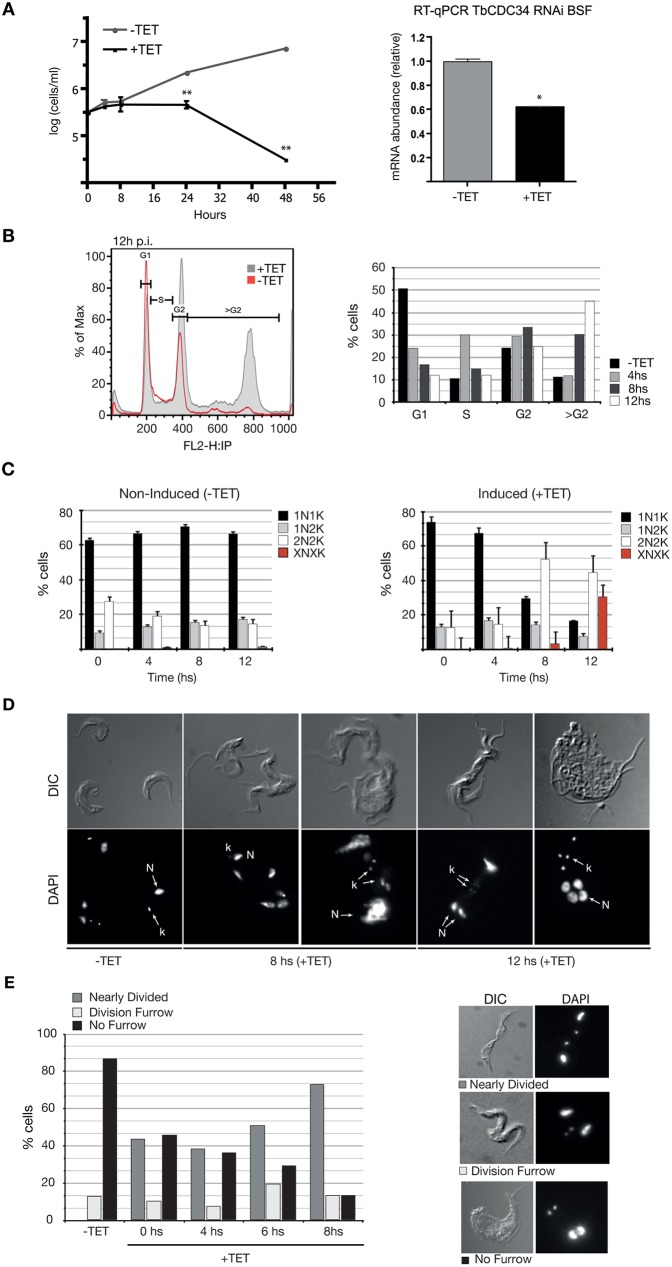
TbCDC34 is necessary for cytokinesis in BSF *T*. *brucei*. (A) Growth curve of BSF TbCDC34-RNAi parasites. Parasites were transfected with pZJM-TbCDC34-RNAi construct. Cells were cultured in the absence (-TET) or presence (+TET) of tetracycline (1μg/ml) (left panel). RT-qPCR demonstrating the RNAi-mediated down regulation of TbCDC34 (right panel). Error bars represent the ± SEM of 3 biological replicates. *: *p≤ 0*.*01;* **: *p≤ 0*.*001*. (B) Flow cytometry analysis of TbCDC34-RNAi BSF parasites. The parental 90–13 cell line is shown as control. The x-axis shows fluorescence intensity in the FL2-A channel. The right panel indicates percentage of cells in different cell cycle stages as determined using the FlowJo 7.5.5 software. (C) Nucleus and kinetoplast configurations in non-induced (-TET) cells (left) and RNAi- induced (+TET) cells (right). At least 200 cells were counted for each time point. (D) Images of DAPI-stained BSF cells viewed by DIC and fluorescence microscopy. The effect of TbCDC34 downregulation on cellular morphology, the nucleus (N) and the kinetoplast (K) is observed. E) Characterization of cytokinesis stage of 2N2K cells from TbCDC34-RNAi cell line cultured in the presence or absence of tetracycline (TET) at different time points. 90–13 is the parental cell line used to generate the RNAi cell line.

**Fig 4:**
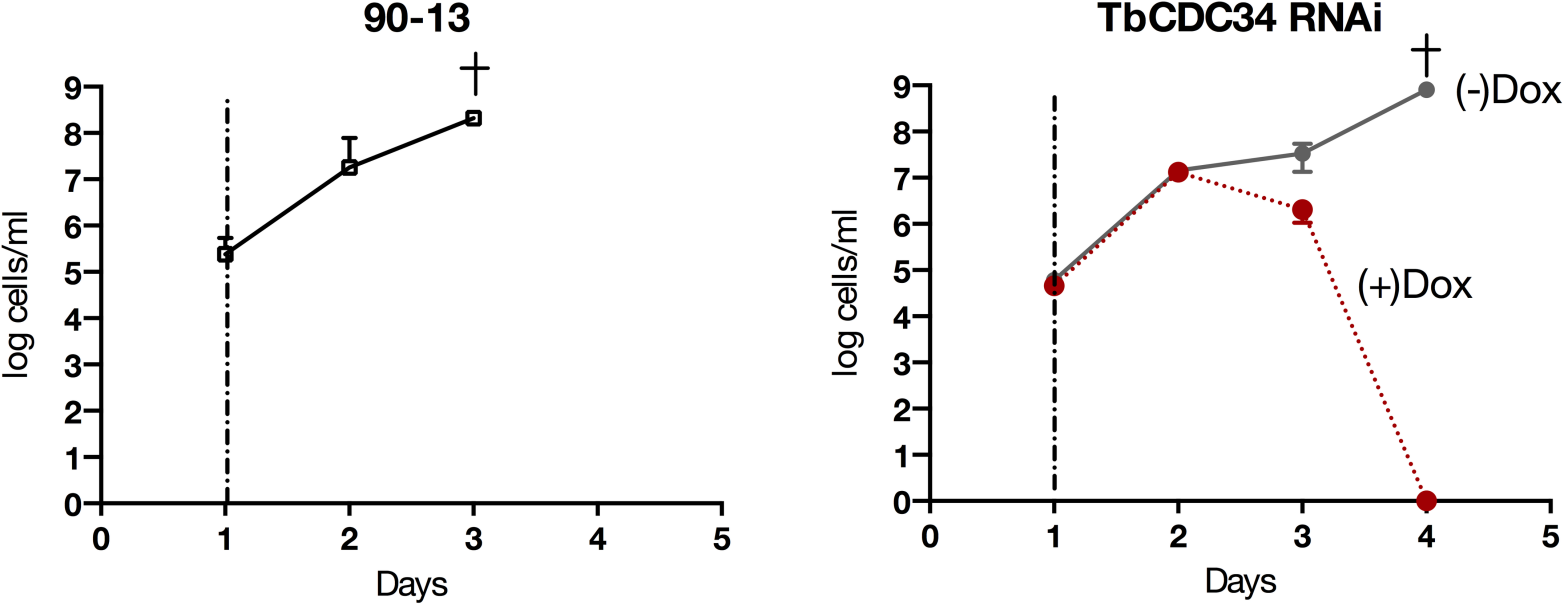
TbCDC34 is necessary for infection progression in mice. Parasitemia was monitored in peripheral blood from one to four days after intraperitoneal inoculation of parental BSF *T*. *brucei* cell line 90–13 (left) or TbCDC34-RNAi BSF *T*. *brucei* cells (right). Dashed vertical lines indicate the times at which doxycycline was added to the drinking water of the selected animals. Application of doxycycline to the TbCDC34-RNAi cell line (+Dox, *right panel*) resulted in clearance of trypanosomes from mice. This did not occur in mice not given doxycycline (-Dox, *right panel*). These results are representative of at least three experiments. Crosses (✝) indicate that the mice reached a humane end point within 24 h of the last recorded parasitemias. Error bars represent the ± SEM of 5 biological replicates.

To determine whether the identified *T*. *brucei* SCFC homologue genes have a role in the regulation of trypanosome cell cycle, procyclic and bloodstream parasite forms were transfected with constructs for tetracycline-inducible RNAi against TbRBX1, TbSKP1, TbCULLIN1 and TbCDC34. After tetracycline induction, the abundance of targeted gene transcripts was determined and the effects on proliferation and on the cell cycle of *T*. *brucei* were examined. While knockdown of TbSKP1, TbRBX1 and TbCDC34 led to slower population growth rates (Figs 1–4), downregulation of TbCULLIN1 failed to register any detectable effect on the growth of both life forms of *T*. *brucei* (S5 Fig).

#### TbRBX1 depletion influences kDNA replication

Depletion of TbRBX1 resulted in a noticeable slowdown in PCF growth rate four days after induction of RNAi (Fig 1A). In contrast, no detectable effect was observed on the growth of BSF parasites (Fig 1A, *right panel*), although northern blot and RT-qPCR showed a decrease in the *TbRBX1* mRNA upon the induction. Flow cytometry analyses showed a reduction in the percentage of G1 cells (13% decrease) by day five after RNAi induction, with no change in S phase and a slight increase in the G2 population (5%) (Fig 1B). We also analyzed the position of cells in cell cycle by DAPI staining (4,6-diamidino-2-phenylindole). Cells that are in the G1 phase of the cell cycle contain one nucleus and one kinetoplast (1N1K) and G2/M cells contain one nucleus and two kinetoplast (1N2K), while postmitotic cell have 2 nuclei and 2 kinetoplasts (2K2N) [25, 26]. By day six after PCF RNAi induction, DAPI analyses showed a reduction in the percentage of 1N1K cells in the TbRBX1 depleted cells compared to the non-induced control cells (88% vs. 71%). By day seven, this difference was further increased (86% vs. 68%) (Fig 1C). There were no obvious alterations in the number of either 1N2K or 2N2K cells, but by day six after induction, the number of aberrant cells with 1N0K and 0N1K (zoids) increased (11.8% and 3.4%, respectively) (Fig 1C, *right panel* and 1D). No appreciable changes in cell morphology were observed in the time period studied. These results suggest that TbRBX1 could be involved in the replication of the kinetoplast, given that its depletion produces the appearance of cells with one nucleus and no kinetoplast, and cells with one kinetoplast and no nucleus (zoids).

#### TbSKP1 promotes progression through the G1/S transition of the *T*. *brucei* cell cycle

We next analyzed the effect of downregulating TbSKP1 by RNAi in three different clones of both BSF and PCF lines. In PCF cells, induction of TbSKP1 RNAi led to a slower parasite growth rate, which was evident after four days of tetracycline induction. Cessation of BSF growth occurred 24 hours after induction (Fig 2, *right panel*). Downregulation of *TbSKP1* mRNA was confirmed by Northern blot (for PCF) and RT-qPCR (for BSF) (Fig 2A). When examined by flow cytometry, TbSKP1-depleted PCF cells showed a 12% increase in G1/S cells after six days of RNAi induction, while cells in G2/M and S-phase configuration had an 8% and 9% decrease, respectively (Fig 2B, *left panel*). Similar results were found in TbSKP1-depleted BSF cells: 14.3% increase in G1/S cells, 6.8% decrease in S-phase cells and 2.4% decrease in G2/M cells compared to the non-induced controls (Fig 2B, *right panel*). Differences in DAPI-stained cells were subtler (this could be due to the fact that G1 and S-phase *T*. *brucei* cells are difficult to distinguish by nuclear and kinetoplast configuration analysis); nonetheless, the majority of the tetracycline-induced cells were in the G1/S phase of the cell cycle (Fig 2C) and as an example, some are shown in Fig 2D. The enrichment of G1/S cells in both forms of *T*. *brucei* when TbSKP1 is depleted suggests that this protein could have a similar function to its homologues in yeast and humans, namely the ubiquitination of cell cycle regulators at the transition from the G1 to the S phase of the cell cycle [27, 28].

#### TbCDC34 is necessary for cytokinesis

Downregulation of TbCDC34 was only analyzed in BSF parasites; no stable cell lines were obtained for PCF cells after several transfection attempts, suggesting that leakiness of the construct might be deleterious. In BSF, induction of TbCDC34 RNAi resulted in a rapid growth arrest, which was evident after 8 hours of tetracycline induction (Fig 3A, left panel), and correlated with the decrease in TbCDC34 mRNA levels (Fig 3A, right panel). Flow cytometry analysis showed an increase in cells with 4C DNA content and the appearance of tetraploid cells (8C) after 8 h of RNAi induction (Fig 3B, left panel). A significant increase in post-mitotic (2N2K) cells was observed over time by DAPI staining (n > 300 cells), with these cells comprising 50% of the cells at 8 h after tetracycline induction (Fig 3C, right panel). We observed cells that remained joined at their posterior ends (Fig 3D). These cells went on to re-duplicate their nuclear and kinetoplast DNA, resulting in the appearance of abnormal cell types over time, with 30% of the cells having abnormal nucleus/kinetoplast (XNXK) configurations at 12 h post tetracycline induction (Fig 3D). After this time point, almost no viable cells remained in the culture. These cells seemed to display a partially ingressed cleavage furrow or an incomplete abscission of the cells (Fig 3D). Non-induced cells, although growing at slower rate compared to the wild-type cells, did not show these abnormalities (Fig 1D, -TET). Further examination of tetracycline-treated cells revealed that 75% of the 2N2K cells were in the final stages of cytokinesis (abscission), 17% had not yet commenced cytokinesis furrowing and around 15% were in the process of furrowing (Fig 3E). These results suggest that one or more substrates of TbCDC34 have a major role on cell abscission in BSF T. brucei cells.

#### TbCDC34 is necessary for *T*. *brucei* infection progression *in vivo*

To determine whether TbCDC34 is also essential for infection **progression** in a mammalian host [29], C3H/He mice were inoculated with BSF TbCDC34-RNAi cells. The parental *T*. *brucei* 90–13 strain from which the RNAi clones were derived was used as a control. Mice were either untreated or treated with 1 mg/ml of doxycycline added to the drinking water in order to induce RNAi *in vivo* and parasitemia was quantified in peripheral tail blood from one to four days after doxycycline induction. When mice exhibited a noticeable parasitemia (~5x10^7^ cells/ml), doxycycline (1 mg/ml) was added to the drinking water of five mice. In mice not provided with doxycycline, a humane end point was reached within 96 h (Fig 4, *left panel*). In contrast, addition of doxycycline resulted in a rapid clearance of circulating parasites even at high parasitemia (Fig 4, *right panel*). The failure of parasites to progress in an infection with TbCDC34 RNAi might have been predicted from *in vitro* assays. However, demonstration in an *in vivo* model of infection is a significant and necessary validation of the key role of TbCDC34 in infection.

### Overexpression of TbCDC34

**Fig 5:**
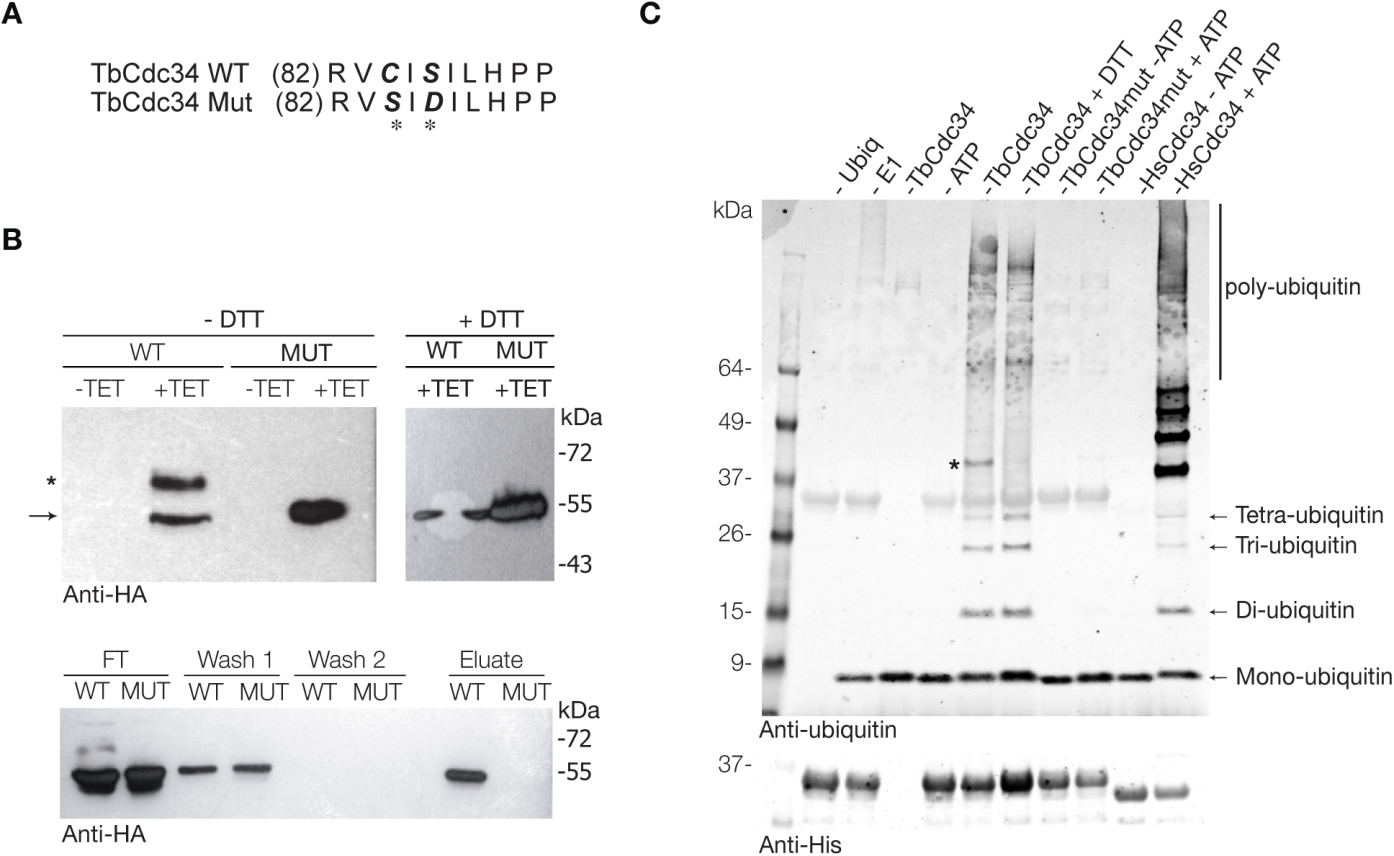
Overexpression of wild-type and mutant TbCDC34-6HA proteins. (A) Schematic representation showing the wild type (WT) version of TbCDC34 and the amino acids mutated in TbCDC34MUT. (B) Western blot analysis after induction of overexpression of WT and double mutant TbCDC34 with a C-terminal 6HA tag in PCF *T*. *brucei* cells. Same amount of parasites were lysed under non-reducing (-DTT) or reducing (+DTT) conditions (upper panel). In the absence of DTT, a higher molecular species of TbCDC34 is observed. In the presence of DTT, this species was no longer detected (+DTT, right panel). The arrow indicates the band corresponding to the expected size of TbCDC34-6HA and the asterisk indicates a higher molecular band only detected in the WT protein in absence of reducing agent. Extracts from WT or mutant TbCDC34 overexpressing parasites were passed through an ubiquitin capture matrix and analyzed by western blot (bottom panel). (C) In vitro ubiquitination assay. Recombinant WT and mutant His6-TbCDC34 or His6-HsCDC34 (10 μM) were incubated with 100 nM of human E1 and 5 μM of human ubiquitin for 3 hours at 37°C, in the presence (+ATP) or absence (-ATP) of ATP. Controls include reactions without ubiquitin (-Ubiq), without E1 (-E1) or without TbCDC34 (-TbCDC34). Reactions were stopped in SDS sample buffer with or without DTT and analyzed by SDS-PAGE 4–12% gels and western blotting with anti-ubiquitin and anti-His antibodies. The asterisk indicates the thioester adduct (TbCDC34-Ub). Unspecifically detected TbCDC34 is observed in the anti-ubiquitin blot.

In *S*. *cerevisiae*, the double mutant *CDC34-C95S*,*L99S* encodes an inactive ubiquitin-conjugating enzyme that blocks cell growth when overexpressed in wild-type strains [22, 30]. In order to investigate the effect of overexpressing TbCDC34 in *T*. *brucei*, full-length and a double mutant HA-tagged versions of the protein were expressed under the control of the tetracycline repressor in procyclic and bloodstream form cells (CDC34-6HA and CDC34^MUT^-6HA) [16]. Site-directed mutagenesis was used to generate a mutant version of the putative ubiquitin-accepting amino acids Cys84 (C84S) and Ser86 (S86D) of TbCDC34. Growth curves of parasites overexpressing TbCDC34 or TbCDC34^MUT^ showed no obvious differences when compared to their non-induced controls, either in PCF or in BSF cells. Protein expression was confirmed by western blot with anti-HA antibody (Fig 5B).

Besides the protein corresponding to the tagged version of TbCDC34, a second higher molecular mass protein was observed only in the extracts of the induced parasites transformed with the wild-type version of the protein, but not in the extracts with the double mutant. This higher molecular mass protein was no longer detected in the presence of dithiothreitol (DTT) (Fig 5B). Since the formation of E2~ubiquitin thioester can be monitored as an 8 kDa SDS–PAGE mobility shift under non-reducing conditions, we concluded that this DTT-sensitive band corresponds to TbCDC34 charged with ubiquitin. To confirm this further, we performed immunoprecipitation assays against the HA epitope in *T*. *brucei* extracts overexpressing TbCDC34-6HA and TbCDC34^MUT^-6HA using a matrix that isolates both mono- and poly-ubiquitinated proteins. TbCDC34 proteins were then analyzed by western blot with anti-HA antibody. Flow through analysis showed that the majority of the higher molecular mass species in the wild-type TbCDC34-expressing sample were retained on the matrix (compare upper and lower panels in Fig 5B), while the unmodified wild type and double mutant proteins were not retained. TbCDC34-6HA but not the TbCDC34^MUT^-6HA version eluted from the matrix (Fig 5B), although the DTT-sensitive conjugate was not detected, likely due to the release of the thioester during elution procedure.

To ensure that TbCDC34 can indeed conjugate ubiquitin molecules, we performed an *in vitro* gel-based ubiquitination assay. This assay employed purified recombinant wild-type His6-TbCDC34, His6-TbCDC34^MUT^ or human His6-HsCDC34 as a positive control, together with human ubiquitin and the ubiquitin-activating enzyme (Ube1) (human ubiquitin has only three amino acid differences to *T*. *brucei* ubiquitin and can be used as a substrate). Western blotting using anti-ubiquitin antibody revealed that the wild-type TbCDC34, but not the mutant TbCDC34 conjugated human ubiquitin molecules and autoubiquitinated in an ATP-dependent manner. We detected an ubiquitination ‘ladder’ of higher molecular weight protein species corresponding to the ubiquitinated TbCDC34 or free poly-ubiquitin chains in a similar manner than for HsCDC34, although with a weaker signal intensity (Fig 5C), likely reflecting a lower enzyme specificity to the human ubiquitin and the human E1 enzyme. Moreover, like the human CDC34 protein [31], TbCDC34 was able to produce di-, tri- and tetra-ubiquitin (Fig 5C).

### TbCDC34 localization and cell cycle expression

**Fig 6:**
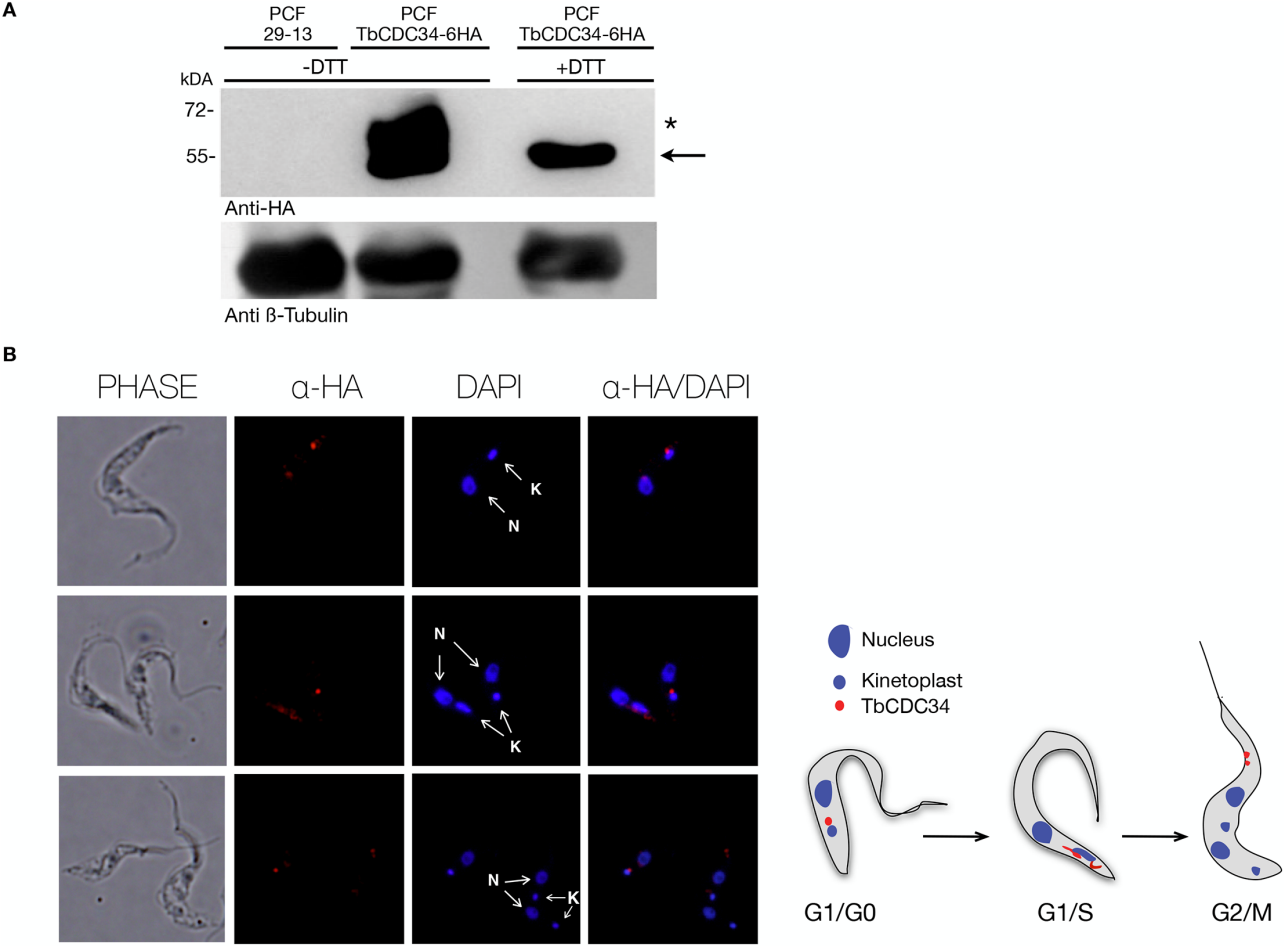
Endogenous TbCDC34-6HA localization in PCF *T*. *brucei*. (A) Western blot showing TbCDC34-6HA expression. PCF 29–13 cells harboring pENT6B-TbCDC34-6HA in one of the two alleles of the TbCDC34 genomic locus were used to examine the endogenous TbCDC34-6HA expression. Wild-type PCF 29–13 cells were used as negative controls (lane 1). Total proteins from 5x10^6^ cells were analyzed by western blot with anti-HA antibody (top) or anti-β-tubulin antibody as a loading control (bottom). The arrow indicates the band corresponding to the expected size of TbCDC34-6HA and the asterisk indicates a higher molecular band only detected in absence of reducing agents. (B) Indirect immunofluorescence of endogenous TbCDC34-6HA using anti-HA antibody. The 29–13 cells with the endogenous TbCDC34-6HA harboring pENT6B-TbCDC34-6HA construct were fixed with 4% paraformaldehyde and stained with primary anti-HA antibody and secondary anti-rabbit Alexa Fluor 546 (red) and DAPI (blue). N: *Nucleus*, K: *kinetoplast*.

The subcellular localization of TbCDC34 was investigated by immunofluorescence. A procyclic cell line expressing TbCDC34-6HA from the endogenous locus was generated, and expression of TbCDC34-6HA was confirmed by western blot (Fig 6A). Immunostaining of the endogenously expressed TbCDC34-6HA protein in PCF cells indicated that it localized near the kinetoplast, in a specific spot between the nucleus and the kinetoplast (Fig 6B). This specific localization was only seen in cells that were in the G1/S window, as judged by DAPI analysis. When the kinetoplast started duplication, this spot disappeared and the signal became diffuse in the cytoplasm (Fig 6B). In some cells, TbCDC34-6HA was localized in foci at the anterior tip of the cell.

**Fig 7:**
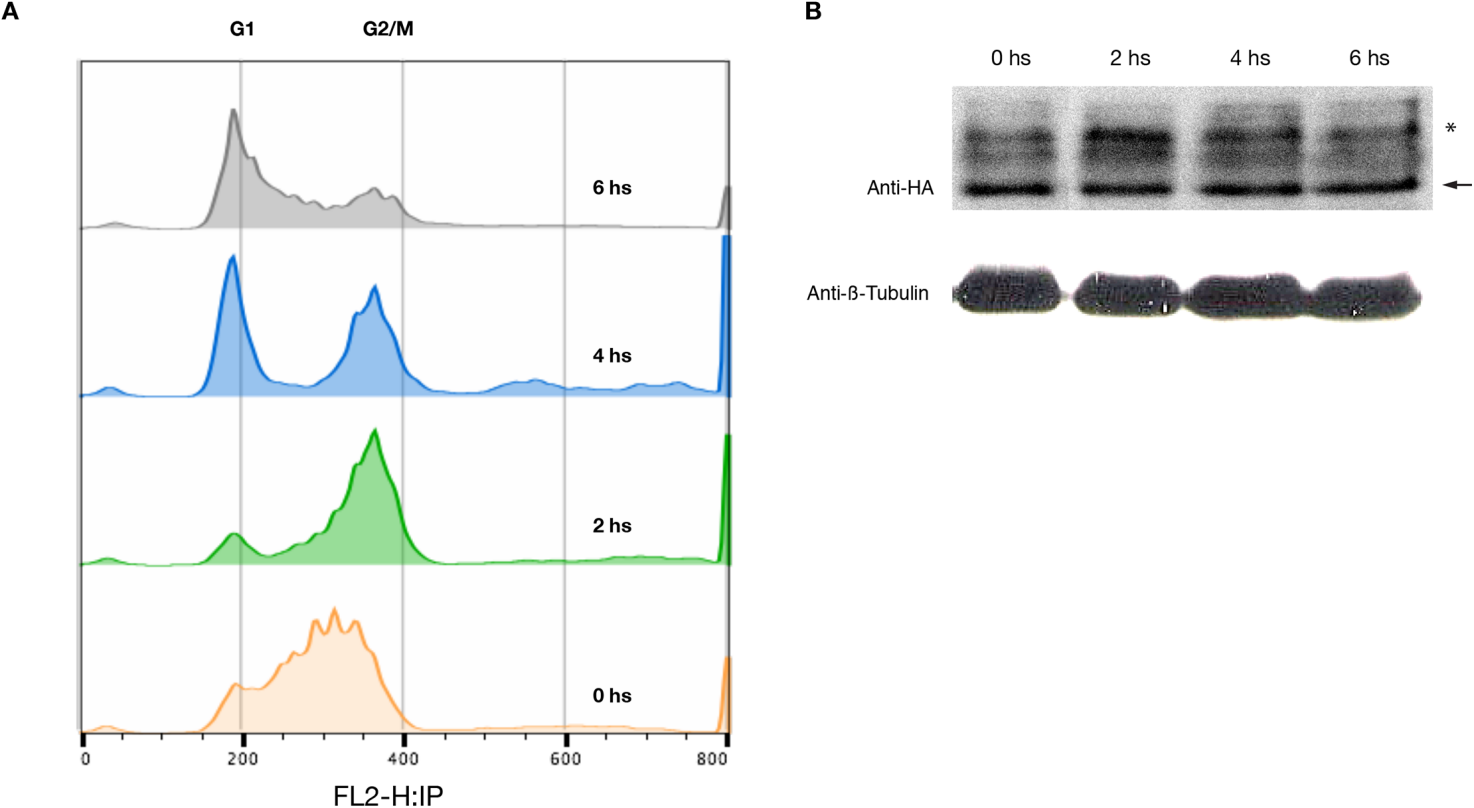
Hydroxyurea synchronization of PCF cells expressing endogenous tagged TbCDC34-6HA. PCF 29–13 cells harboring pENT6B-TbCDC34-6HA were treated for 12 h with 0.2 mM hydroxyurea (HU), washed twice, and culturing was resume without HU. Samples were taken every two hours for flow cytometry and western blot analysis. (A) Histograms indicate results from cell sorting for DNA contents after cell cycle synchronization (propidium iodide channel). Dashed lines indicate the peak locations in the histograms from cell cycle. (B) Western blot showing protein levels of TbCDC34-6HA after HU release. Total proteins from 5x10^6^ cells were stained with anti-HA antibody (top) or anti-β Tubulin as a loading control (bottom). The loading buffer did not contain DTT. Arrow indicates the band corresponding to TbCDC34-6HA and the asterisk indicates the band corresponding to the modified version of the protein.

Next, we investigated if the levels of the TbCDC34 protein changed during the cell cycle of the parasites. For this purpose, we synchronized with hydroxyurea PCF cells expressing TbCDC34-6HA from the endogenous locus [32]. Parasites were cultured for 12 hours in the presence of 0.2 mM hydroxyurea, washed, and the culture was allowed to resume growth in the absence of hydroxyurea. Cell samples were collected once every 2 hours and subjected to flow cytometry analysis and western blot. At the moment when hydroxyurea was removed, most of the cells were synchronized at S phase of the cell cycle; by 2 h they shifted to the G2/M phase. From 4 to 6 h after hydroxyurea removal, there was a constant shift from the G2/M phase to the G1 phase (Fig 7A). Synchronized samples were analyzed by western blot using the anti-HA antibody for quantification of TbCDC34-6HA, in the absence of DTT to analyze the levels of the tagged protein and possible changes in the posttranslational modification of TbCDC34. The TbCDC34 protein levels remained constant throughout the cell cycle, although a slight increase of the modified version of the protein could be observed at 2 h after hydroxyurea removal (G2/M) (Fig 7B).

### TbCDC34 activity is affected by compound CC0651

**Fig 8:**
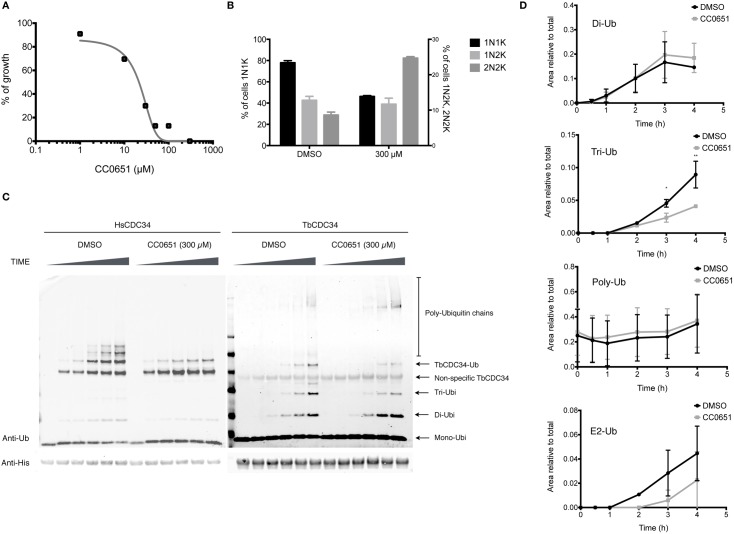
TbCDC34 activity is modulated by compound CC0651. (A) BSF cells were incubated with different concentrations of CC0651 and growth inhibition was calculated after 48 hours of culture. (B) Cells treated or untreated were fixed and analyzed for DNA content using DAPI. Samples treated with CC0651 showed a decrease of cells with 1N1K and an increase of cells with 2N2K. (C) *In vitro* ubiquitination assay. Recombinant HsCDC34 (left panel) or TbCDC34 (right panel) (10 μM) was incubated with 100 nM of human E1 and 5 μM of human ubiquitin for 0, 1, 2, 3 and 4 hours at 37°C, in the presence of 300 μM of CC0651 or DMSO. Reactions were stopped in SDS sample buffer without DTT and analyzed by SDS-PAGE 4–12% gels and western blotting with anti-Ubiquitin and anti-His antibodies (D) Changes in the levels of Mono, Di, Tri or Poly-ubiquitin and TbCDC34~Ub thioester formation were quantified and compared between treatments. Values are means ± SEM of 3 individual samples. *: *p≤ 0*.*02;* **: *p≤ 0*.*001*.

So far, the small-molecule termed CC0651 is the only known selective inhibitor of the human CDC34 and one of very few described E2 enzyme inhibitors. CC0651 inserts into a cryptic binding pocket on hCDC34, causing a displacement of E2 secondary structural elements of CDC34 [33]. In order to determine if CC0651 has an effect on TbCDC34 and if it could be used to inhibit *T*. *brucei* growth, we performed growth-inhibition assays with different concentrations of the compound. CC0651 inhibited cell growth with an IC50 of 21.38 μM (Fig 8A), which is similar to the reported IC50 for PC-3 cells [33]. However, in our assay, we measured the growth inhibition at 48 hours, as compared to 5 days for the human cells [33]. Next, we analyzed the effect of the compound on the cell cycle of the parasites. As shown in Fig 8B, there was a decrease of cells in the G1 phase (77.9% to 46.3%), an increase of cells in G2/M phases (8.6% to 24.8%) and an increase in aberrant cells XNXK (0.4% to 17%), with no apparent changes in S phase. These results are similar to the effect observed in the TbCDC34 RNAi experiments, indicating that the compound may act through inhibition of the trypanosomal enzyme. To corroborate that the compound is acting upon TbCDC34, we performed an *in vitro* gel-based ubiquitination assay in the presence or absence of CC0651. As a control, the reaction was also performed with the human CDC34 (Fig 8C, left panel). The addition of CC0651 to the reaction had modest, but detectable effect on TbCDC34 activity: production of free tri-ubiquitin was significantly decreased at later time points (Fig 8D), while the production of di-ubiquitin increased slightly, but not significantly, and no significant effect was observed on the formation of the thioester adduct (TbCDC34~Ub) and of the assembly of polyubiquitin chains. These results resemble the mild effects of the compound CC0651 on the human CDC34 [33] and indicate that TbCDC34 activity is modulated by compound CC0651 *in vitro* and potentially *in culture*, as a similar phenotype is observed when treating cells with the compound and the RNAi against TbCDC34.

## Discussion

In this study, we have selectively down-regulated the expression of the core SCFC subunit homologs and the E2 enzyme CDC34 by RNAi in both the procyclic and bloodstream forms of *T*. *brucei*. Contrary to what was reported for yeast and human cells (i.e. necessary for the G1/S transition), downregulation of TbSKP1, TbRBX1, TbCULLIN1 and TbCDC34 generated different phenotypes that would indicate that they might not be assemble in a stable complex.

Downregulation of TbRBX1 affected the parasite’s growth in the procyclic but not in the bloodstream form (Fig 3A). DNA content analysis of TbRBX1-depleted cells showed a rise in the percentage of cells without kinetoplast, with no changes being observed in nuclear DNA (Fig 3C and 3D). When analyzed by flow cytometry, a pronounced decrease in the number of cells at the G1 stage of the cell cycle was observed (Fig 3B). These results might indicate that TbRBX1 functions in the maintenance or replication of the kinetoplast DNA in procyclic cells.

Downregulation of TbSKP1 resulted in an increase of cells in the G1/S transition both in the procyclic and the bloodstream form, consistent with what was observed in *H*. *sapiens* and *S*. *cerevisiae* [5, 27, 34, 35]. In *S*. *cerevisiae*, SKP1 mutants arrest in G1 with a 1C DNA content or in G2, indicating that SKP1 is required for S phase entry and mitosis [27]. It was reported that downregulation of TbSKP1 resulted in a G2/M blockade in the bloodstream form trypanosomes [36]. The different phenotypes observed in our and Benz and Clayton’s work could be attributed to the different RNAi plasmids employed and unavailability of information on remaining protein levels. Our results show a similar RNAi-induced phenotype in both life cycle stages of *T*. *brucei*. Nevertheless, we cannot rule out that TbSKP1 could have functions in both G1 and G2/M cell cycle checkpoints, as it has been previously shown in other eukaryotic cells. The SKP1 acts at different cell cycle checkpoints by binding to different F-box proteins and recognizing different phosphorylated substrates [35].

The lack of a detectable phenotype for TbCULLIN1 could be attributable to expression of proteins with redundant function. Indeed, there are several coding genes for the cullin family in the parasite’s genome. We identified at least seven genes with cullin-homology domains in *T*. *brucei* (Tb927.8.5970, Tb927.11.11430: similar to HsCULLIN1; Tb927.10.7490: similar to HsCULLIN2; Tb927.3.1290: similar to HsCULLIN4b; Tb927.8.5210: similar to CULLIN3; and Tb927.10.6930 and Tb10.v4.0249: with no similarity to any known cullin). Taking these data into account, it is possible that another family member could compensate the depletion of TbCULLIN1. Also we cannot exclude the possibility that Tb927.8.5970 analyzed in this study is not orthologous gene of CULLIN1.

The strongest and clearer phenotype was observed with TbCDC34 knockdown. The decreased number of cells with a 1N1K configuration and the increase in cells with 2N2K and multinucleated/multikinetoplast (Fig 1C) is consistent with a pre-cytokinesis cell cycle arrest. Parasites depleted of TbCDC34 could not complete cytokinesis, but resumed kDNA and nuclear DNA replication, and accumulated as nearly divided 2N2K cells (Fig 1C–1E). Therefore, TbCDC34 activity seems to affect the final stages of daughter cell separation.

The endogenous tagged version of TbCDC34 localized near the kinetoplast in cells that had the configuration 1N1K and, when the kinetoplast started to segregate, TbCDC34 was found in different parts of the cell (Fig 6B). In mammalian cells, it was demonstrated that CDC34/UBE2R1 localizes to punctate structures in interphase cells, predominantly in the nucleus, and in mitotic cells it is recruited to the mitotic spindle at the beginning of anaphase [37]. The different localization of TbCDC34 may reflect differences in function with its human counterpart.

TbCDC34 is able to form a thioester bond with ubiquitin *in vitro* and *in vivo*, and interestingly can accept the activated form of human ubiquitin from the human E1 enzyme, showing the evolutionary conservation of this reaction (Fig 5C). Mutation of Cys84 (C84S) and Ser86 (S86D) of TbCDC34 disrupted the thioester formation, confirming the active site of the enzyme. Addition of the small inhibitor CC0651 to the *in vitro* ubiquitination reaction had modest, but detectable effect on TbCDC34 activity (Fig 8C and 8D), and in culture cells was able to inhibit proliferation of parasites, resulting in a similar cytokinesis defect (Fig 8B).

The depletion of various subunits of the putative SCFC and CDC34 unexpectedly led to different phenotypes, suggesting that they are either involved in the ubiquitination of a number of different proteins regulating the cell cycle or that they associate into different complexes. A search for the exact composition of these putative complexes using tandem affinity protein tag affinity chromatography, may explain the discrepancies encounter in this work. Importantly, our results revealed the essential function of TbCDC34 for the replication of *T*. *brucei*. Effective clearing from infection observed upon knockdown of TbCDC34, the activity inhibition with the small molecule CC0651 (Fig 8) and structural differences between the human and trypanosomal CDC34 which should facilitate development toward specificity, indicate that TbCDC34 could be considered as a potential novel drug target in the African sleeping sickness.

## Supporting information

S1 Fig Amino acid sequences of the putative *T*. *brucei* RBX1 product compared to other eukaryotic RBX1 proteins. The amino acid sequences of RBX1 proteins from *Trypanosoma brucei* (TbRBX1: Tb10.70.6035), *Trypanosoma cruzi* (TcRBX1: Tc00.1047053506495.9), *Leishmania major* (LmRBX1: LmjF21.0023), *Homo sapiens* (HsRBX1: P62877), *Saccharomyces cerevisiae* (ScRBX1: NP_014508), and *Plasmodium falciparum* (PfRBX1: PFC0845c) are aligned. The alignment was generated utilizing ClustalW. Asterisks indicate conserved residues. Numbers indicate residues involved in zinc binding.
(TIF)

S2 Fig Alignment of amino acid sequences of SKP1 proteins from Trypanosoma brucei (TbSKP1: Tb11.02.3990), *Trypanosoma cruzi* (TcSkp1: Tc00.1047053508041.11), *Leishmania major* (LmSkp1: LmjF11.1210), *Homo sapiens* (HsSkp1: P63208), *Saccharomyces cerevisiae* (ScSkp1: P52286), and *Plasmodium falciparum* (Pfskp1: Q8ID38). The alignment was generated utilizing ClustalW. Colored box indicate residues involved in the interaction with the F-box domain of F-box proteins.
(TIF)

S3 Fig Amino acid sequence of the putative *T*. *brucei* CULLIN1 compared to other eukaryotic CULLIN1 proteins. Partial multiple alignment of amino acid sequences of TbCULLIN1 and other eukaryotic homologue proteins. In the upper alignment, the Arg of the cullin-homology (CH) domain whose mutation produces the mutant strain *cdc53-1* in yeast is indicated. In the lower panel, the C-terminal domain of the proteins is shown. The acceptor lysine of NEDD8 in humans and yeast is marked with an arrow. Asterisks denote conservative residues. *Trypanosoma brucei* (TbCul1: Tb927.8.5970), *Trypanosoma cruzi* (TcCul1: Tc00.1047053511075.40), *Leishmania major* (LmCul1: LmjF24.2290), *Homo sapiens* (HsCul1: Q13616), *Saccharomyces cerevisiae* (ScCul1: Q12018), *Plasmodium falciparum* (Pfskp1: PF08_0094).
(TIF)

S4 Fig Amino acid sequence of the putative T. brucei CDC34 compared to other eukaryotic CDC34 proteins. Alignment of the amino acid sequences of CDC34 proteins from *Trypanosoma brucei* (TbCDC34: Tb11.01.5790), *Trypanosoma cruzi* (TcCdc34: Tc00.1047053511727.40), *Leishmania major* (LmCdc34: LmjF32.0960), *Homo sapiens* (HsCdc34: P49427), *Saccharomyces cerevisiae* (ScCdc34: P14682), and *Plasmodium falciparum* (PfCdc34: Q8I301). The alignment was generated utilizing ClustalW. Asterisks indicate conserved residues. Grey residues denote the active site domain. Red residues show the catalytic cysteine residue involved in thioester bond. Blue residues represent the insertion loop, which is a residue segment present only in E2 CDC34 proteins. Black arrows indicate residues phosphorylated in the human or yeast homologue. Half-filled circles indicate residues that could potentially be phosphorylated as predicted using NetPhos 2.0 Server.
(TIF)

S5 Fig Effect of TbCULLIN1 downregulation on *T*. *brucei* growth. Growth curves of PCF TbCULLIN1-RNAi (top panel) and BSF TbCULLIN1-RNAi (bottom panel) parasites after tetracycline induction. PCF or BSF parasites transfected with pZJM-TbCULLIN1-RNAi construct were cultured in the absence (-TET) or presence (+TET) of tetracycline (1μg/ml). Insets in the respective growth curves show northern blot (PCF) or RT-qPCR (BSF) experiments demonstrating the RNAi-mediated downregulation of TbCULLIN1 mRNA. Error bars represent the ± SEM from 3 individual experiments. *: *p≤ 0*.*01*.
(TIF)

S1 Table Primers used in this work for RNAi, overexpression, endogenous tagging and quantitative Real-time PCR. (DOC)
