# Supplementary figures and images for: The ubiquitin-conjugating enzyme CDC34 is essential for cytokinesis in contrast to putative subunits of a SCF complex in Trypanosoma brucei
Source: PLoS Negl Trop Dis. 2017 Jun 13;11(6):e0005626. doi: 10.1371/journal.pntd.0005626 (PMC5507466; doi:10.1371/journal.pntd.0005626)

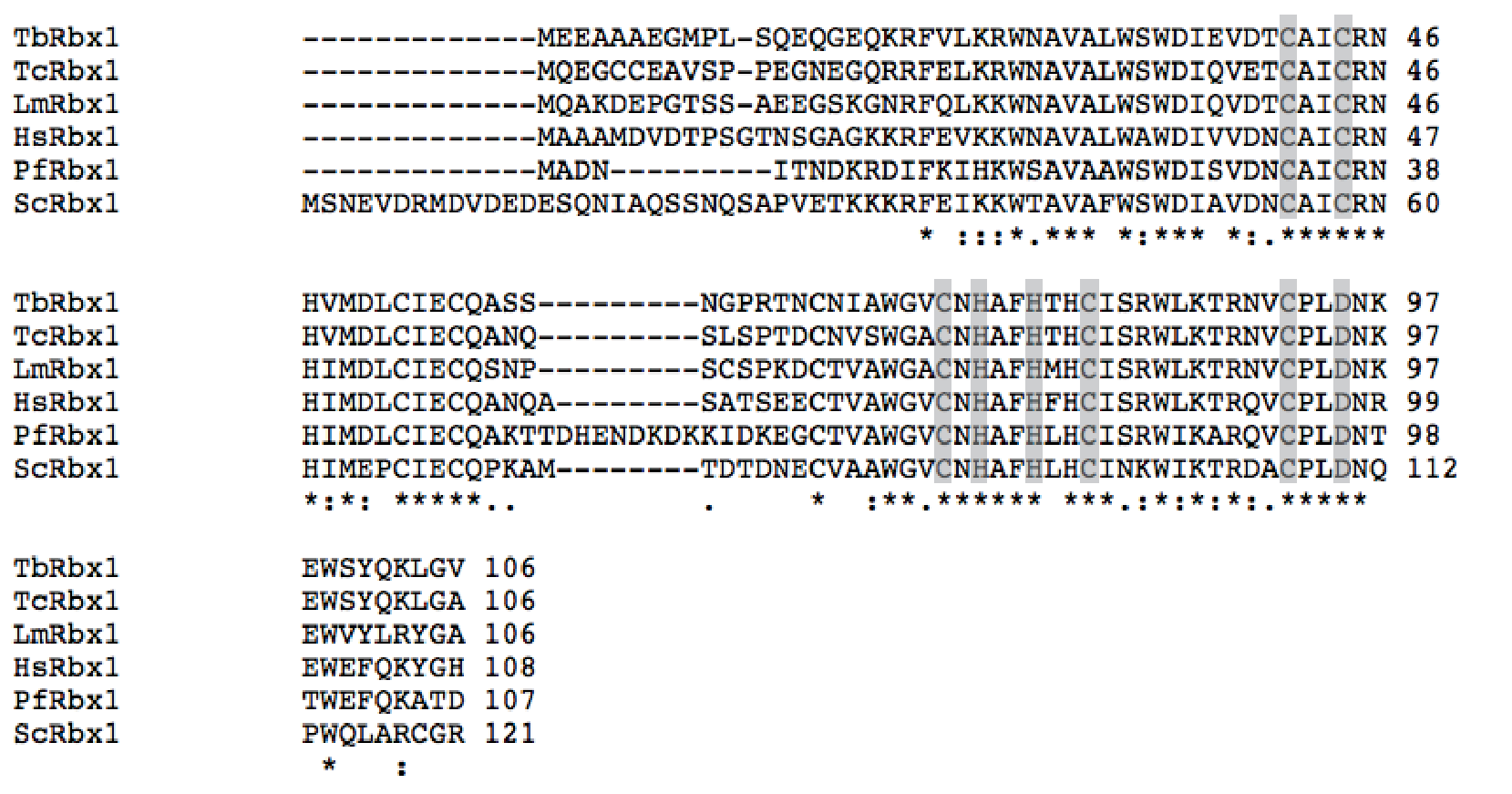

Supplement: S1 Fig — The amino acid sequences of RBX1 proteins from Trypanosoma brucei (TbRBX1: Tb10.70.6035), Trypanosoma cruzi (TcRBX1: Tc00.1047053506495.9), Leishmania major (LmRBX1: LmjF21.0023), Homo sapiens (HsRBX1: P62877), Saccharomyces cerevisiae (ScRBX1: NP_014508), and Plasmodium falciparum (PfRBX1: PFC0845c) are aligned. The alignment was generated utilizing ClustalW. Asterisks indicate conserved residues. Numbers indicate residues involved in zinc binding. (TIF) [file pntd.0005626.s001.tif]

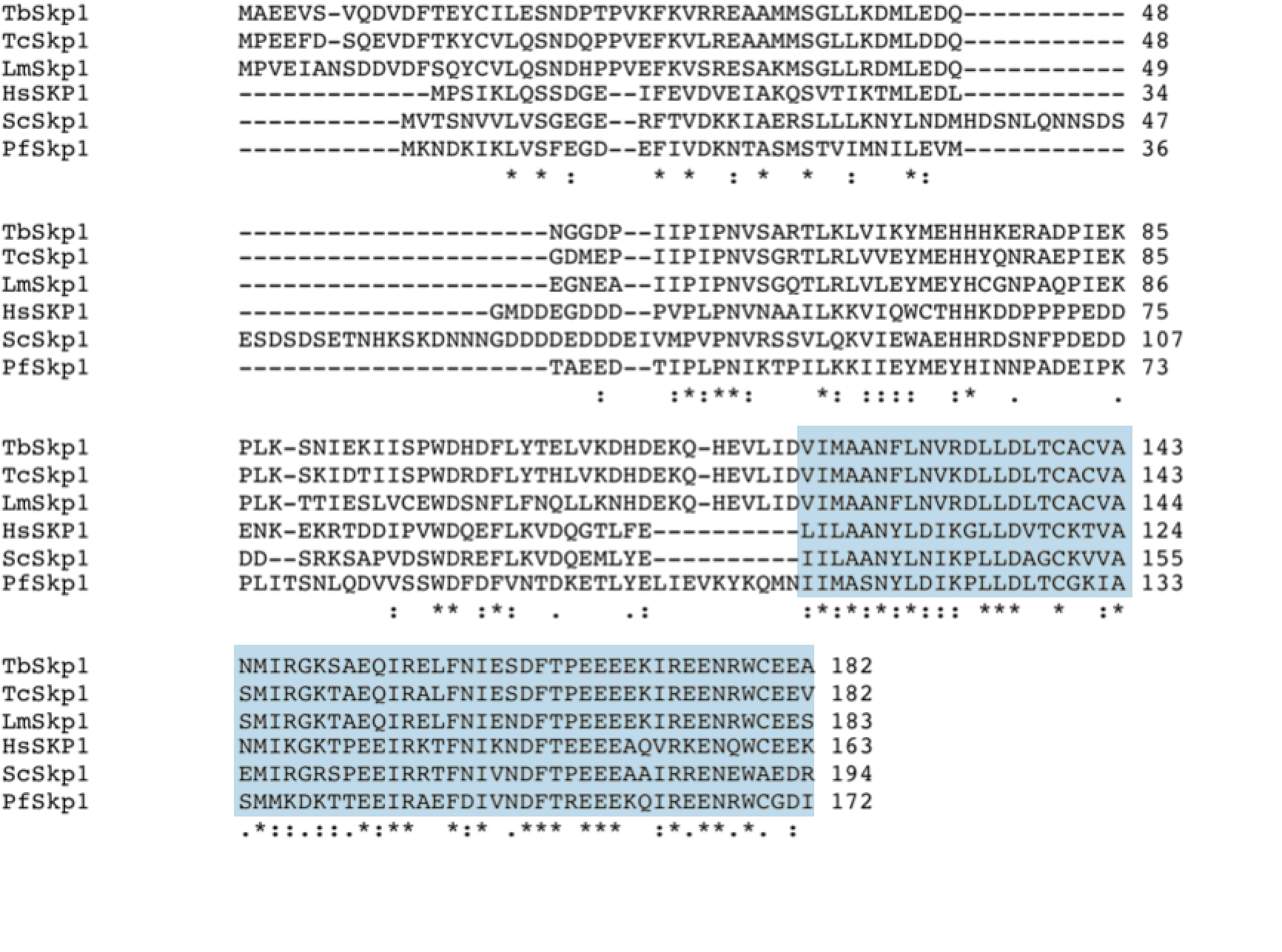

Supplement: S2 Fig — The alignment was generated utilizing ClustalW. Colored box indicate residues involved in the interaction with the F-box domain of F-box proteins. (TIF) [file pntd.0005626.s002.tif]

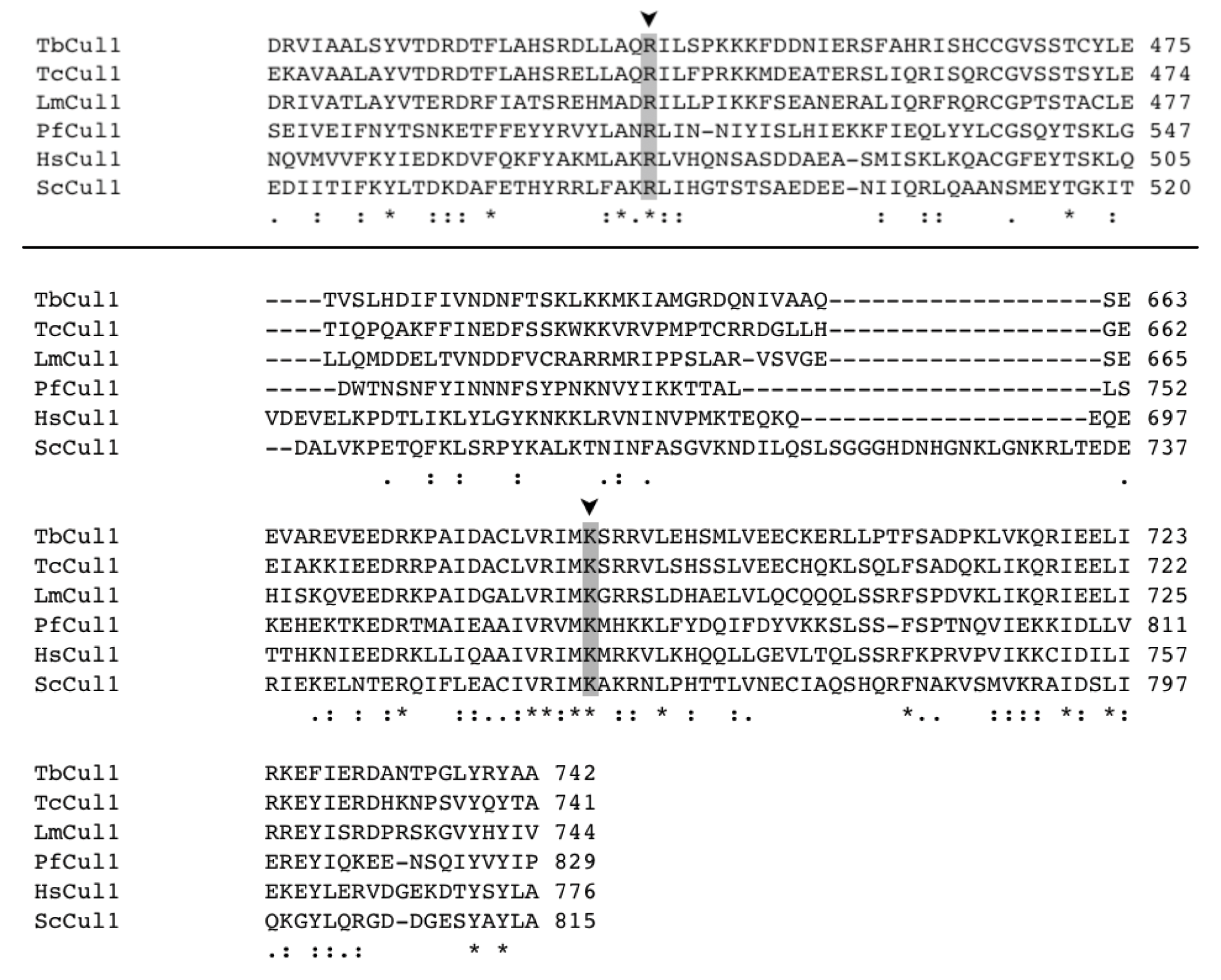

Supplement: S3 Fig — Partial multiple alignment of amino acid sequences of TbCULLIN1 and other eukaryotic homologue proteins. In the upper alignment, the Arg of the cullin-homology (CH) domain whose mutation produces the mutant strain cdc53-1 in yeast is indicated. In the lower panel, the C-terminal domain of the proteins is shown. The acceptor lysine of NEDD8 in humans and yeast is marked with an arrow. Asterisks denote conservative residues. Trypanosoma brucei (TbCul1: Tb927.8.5970), Trypanosoma cruzi (TcCul1: Tc00.1047053511075.40), Leishmania major (LmCul1: LmjF24.2290), Homo sapiens (HsCul1: Q13616), Saccharomyces cerevisiae (ScCul1: Q12018), Plasmodium falciparum (Pfskp1: PF08_0094). (TIF) [file pntd.0005626.s003.tif]

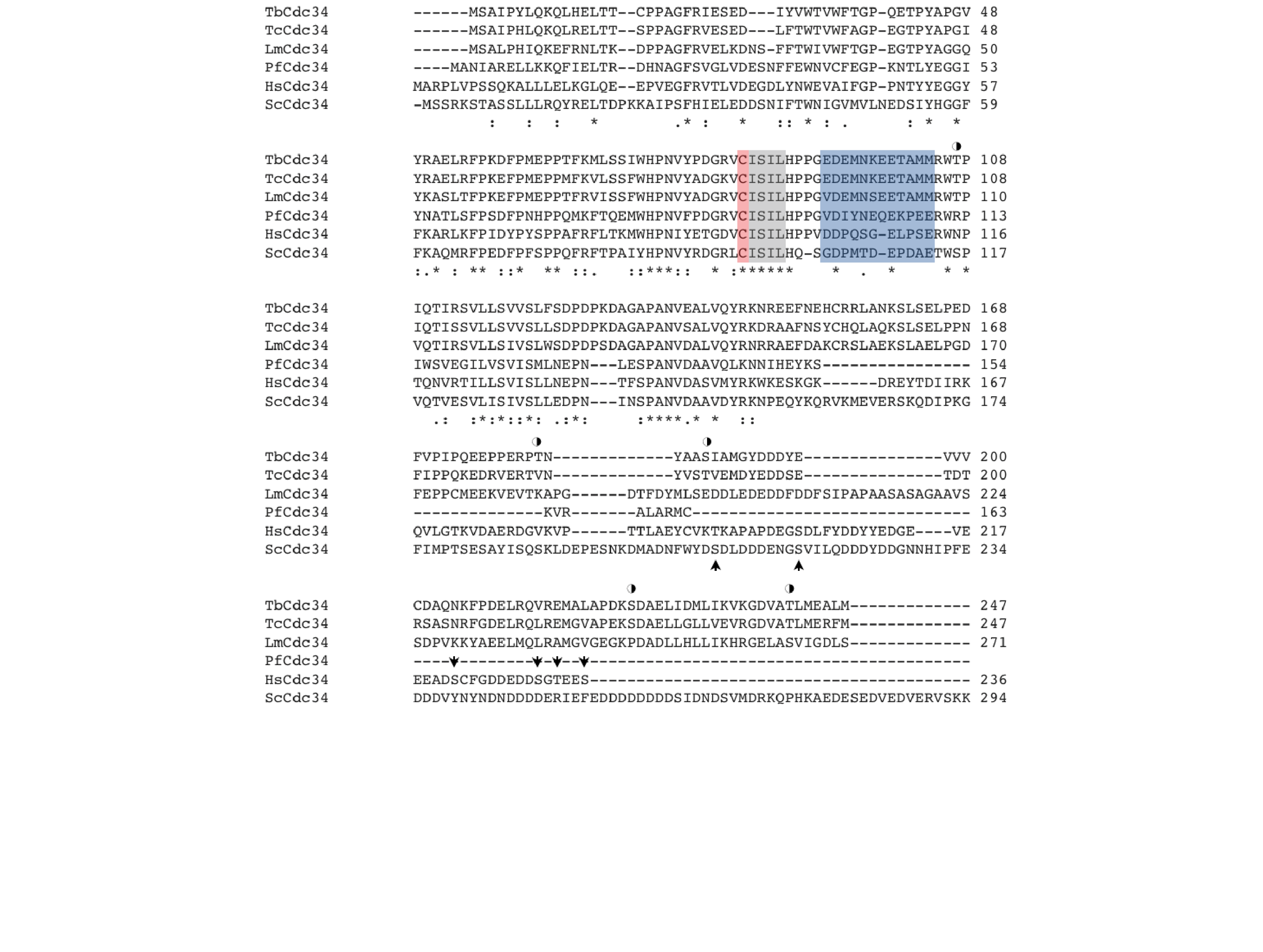

Supplement: S4 Fig — Alignment of the amino acid sequences of CDC34 proteins from Trypanosoma brucei (TbCDC34: Tb11.01.5790), Trypanosoma cruzi (TcCdc34: Tc00.1047053511727.40), Leishmania major (LmCdc34: LmjF32.0960), Homo sapiens (HsCdc34: P49427), Saccharomyces cerevisiae (ScCdc34: P14682), and Plasmodium falciparum (PfCdc34: Q8I301). The alignment was generated utilizing ClustalW. Asterisks indicate conserved residues. Grey residues denote the active site domain. Red residues show the catalytic cysteine residue involved in thioester bond. Blue residues represent the insertion loop, which is a residue segment present only in E2 CDC34 proteins. Black arrows indicate residues phosphorylated in the human or yeast homologue. Half-filled circles indicate residues that could potentially be phosphorylated as predicted using NetPhos 2.0 Server. (TIF) [file pntd.0005626.s004.tif]

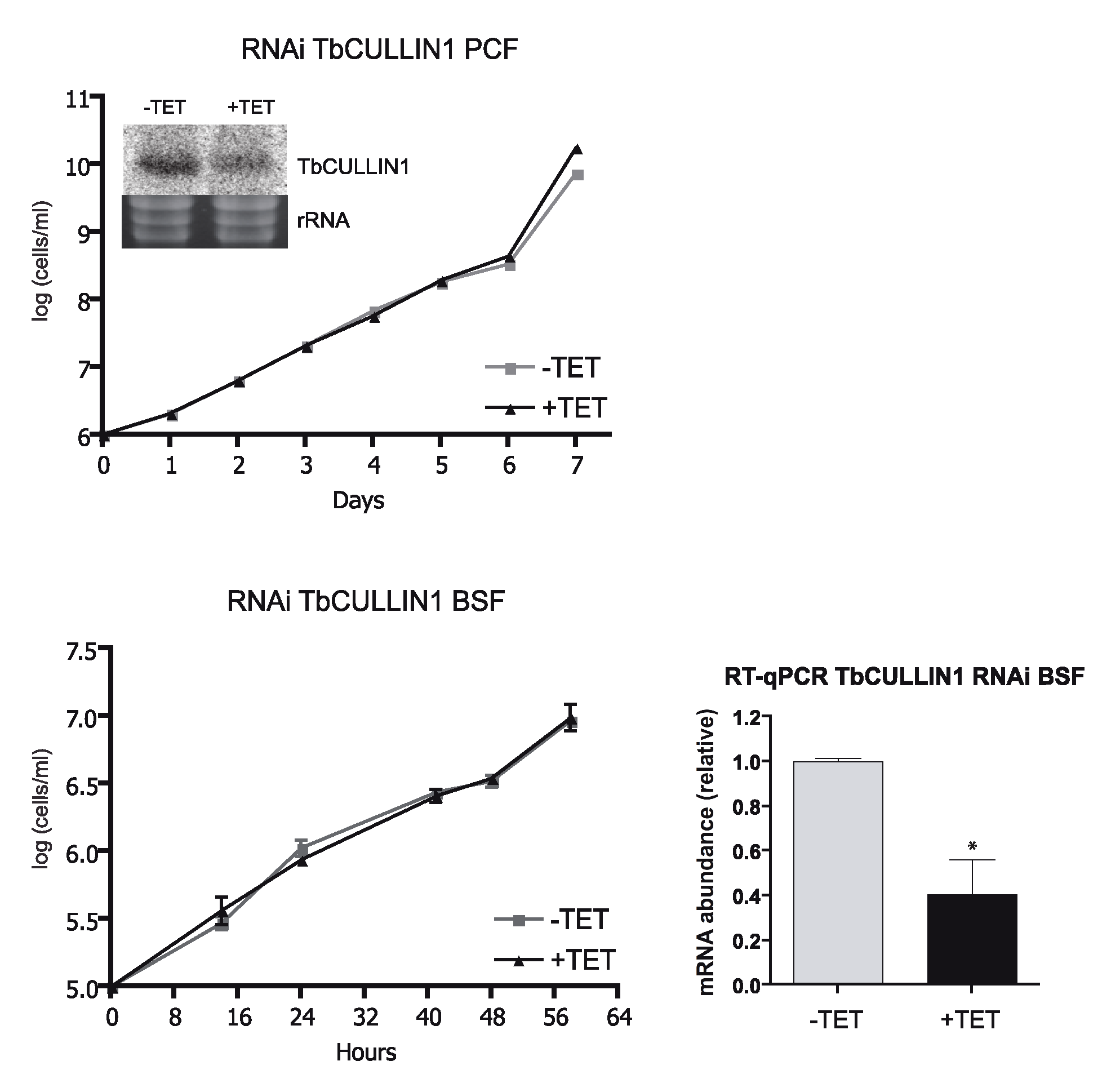

Supplement: S5 Fig — Growth curves of PCF TbCULLIN1-RNAi (top panel) and BSF TbCULLIN1-RNAi (bottom panel) parasites after tetracycline induction. PCF or BSF parasites transfected with pZJM-TbCULLIN1-RNAi construct were cultured in the absence (-TET) or presence (+TET) of tetracycline (1μg/ml). Insets in the respective growth curves show northern blot (PCF) or RT-qPCR (BSF) experiments demonstrating the RNAi-mediated downregulation of TbCULLIN1 mRNA. Error bars represent the ± SEM from 3 individual experiments. *: p≤ 0.01. (TIF) [file pntd.0005626.s005.tif]
