# Supplementary material for: The ubiquitin-conjugating enzyme CDC34 is essential for cytokinesis in contrast to putative subunits of a SCF complex in Trypanosoma brucei
Source: PLoS Negl Trop Dis. 2017 Jun 13;11(6):e0005626. doi: 10.1371/journal.pntd.0005626 (PMC5507466; doi:10.1371/journal.pntd.0005626)
Supplement: S1 Table — (DOC) [file pntd.0005626.s006.doc]

**Table S1. Oligonucleotide sequences.**

**The primers used to perform RNAi constructs were:**

| Primer pairs | Sequence |
| --- | --- |
|  |  |
| pZJM.TbSKP1 |  |
| Fwd: HindIII | cccggatccaagcttcggtacaggacgtgg |
| Rev: XhoI | ccgctcgagcgcacaggtgagatccagta |
| pZJM.TbCDC34 |  |
| Fwd: HindIII | cccggatccaagcttcatgagcttacgtgc |
| Rev: XhoI | ccgctcgagcttcacggttcttgcggta |
| pZJM.TbRBX1 |  |
| Fwd: HindIII | cccggatccaagcttgaggaagcggctgc |
| Rev: XhoI | ccgctcgagttctggtacgaccattcctt |
| pZJM.TbCULLIN1 |  |
| Fwd: HindIII | ccgctcgaggtgtacgctgatggtgttc |
| Rev: XhoI | cgcctcgaaggaatgtaaag |
| **To overexpress proteins in Trypanosomes** | |
| p2477-TbCDC34 |  |
| Fwd (OL2781): HindIII | CCCAAGCTTCCGCCACC ATGTCTGCAATCCCTTAC |
| Rev (OL2780): BamHI | CGGGATCCAGAACCCATCAATGCCTCCATTA |
| **For endogenous tagging in pENT6B**  Fow: HINDIII/XHO AAGCTTccCTCGA GCAGCATTCAACTCTTAC  Rev: SpeI ACTAGT CATAAACCGTTCCATCAG  **To obtain the TbCDC34 mutant:**  CDC34^MUT^ | |
| OL2785 | cccgatggtcgtgtgagtattgatattcttcatccacccggagag |
| **For quantitative realt-time PCR**  Real-Time PCR | |
| SKP1 |  |
| OL2717 | GAACATGATCCGTGGGAAGTCT |
| OL2718 | CGGGAGTGAAGTCGCTTTCA |
| CDC34 |  |
| OL2719 | TTTGTGCCCATTCCTCAAGAG |
| OL2720 | TCGTCATCATACCCCATTGCT |
| CULLIN1 |  |
| OL2721 | GGGCGCTTTGGAATGACA |
| OL2722 | CTAATCAGGATGGATGCCTTCAC |
| RBX1 |  |
| OL2723 | GTTTCTTATTCTCCTCGTTACCTTCAG |
| OL2724 | AAAGAAATCAACCTAGCATATCATAACCT |
| GPI8 |  |
| OL2272 | CGAAGCGCATTTGGATAGC |
| OL2273 | AGCGCGTGATGACAGTGAAG |
